# Metabolic engineering of the oleaginous yeast *Yarrowia lipolytica* PO1f for production of erythritol from glycerol

**DOI:** 10.1186/s13068-021-02039-0

**Published:** 2021-09-25

**Authors:** Sujit Sadashiv Jagtap, Ashwini Ashok Bedekar, Vijay Singh, Yong-Su Jin, Christopher V. Rao

**Affiliations:** 1 Department of Chemical and Biomolecular Engineering, University of Illinois at Urbana-Champaign, Urbana, IL 61801 USA; 2 DOE Center for Advanced Bioenergy and Bioproducts Innovation, University of Illinois at Urbana-Champaign, Urbana, IL 61801 USA; 3 Department of Agricultural and Biological Engineering, University of Illinois at Urbana-Champaign, Urbana, IL 61801 USA; 4 Department of Food Science and Nutrition, University of Illinois at Urbana-Champaign, Urbana, IL 61801 USA

**Keywords:** *Yarrowia lipolytica*, Glycerol, Erythritol, Osmotic stress, Metabolite profiling, Metabolic engineering

## Abstract

**Background:**

Sugar alcohols are widely used as low-calorie sweeteners in the food and pharmaceutical industries. They can also be transformed into platform chemicals. *Yarrowia lipolytica*, an oleaginous yeast, is a promising host for producing many sugar alcohols. In this work, we tested whether heterologous expression of a recently identified sugar alcohol phosphatase (PYP) from *Saccharomyces cerevisiae* would increase sugar alcohol production in *Y. lipolytica*.

**Results:**

*Y. lipolytica* was found natively to produce erythritol, mannitol, and arabitol during growth on glucose, fructose, mannose, and glycerol. Osmotic stress is known to increase sugar alcohol production, and was found to significantly increase erythritol production during growth on glycerol. To better understand erythritol production from glycerol, since it was the most promising sugar alcohol, we measured the expression of key genes and intracellular metabolites. Osmotic stress increased the expression of several key genes in the glycerol catabolic pathway and the pentose phosphate pathway. Analysis of intracellular metabolites revealed that amino acids, sugar alcohols, and polyamines are produced at higher levels in response to osmotic stress. Heterologous overexpression of the sugar alcohol phosphatase increased erythritol production and glycerol utilization in *Y. lipolytica*. We further increased erythritol production by increasing the expression of native glycerol kinase (GK), and transketolase (TKL). This strain was able to produce 27.5 ± 0.7 g/L erythritol from glycerol during batch growth and 58.8 ± 1.68 g/L erythritol during fed-batch growth in shake-flasks experiments. In addition, the glycerol utilization was increased by 2.5-fold. We were also able to demonstrate that this strain efficiently produces erythritol from crude glycerol, a major byproduct of the biodiesel production.

**Conclusions:**

We demonstrated the application of a promising enzyme for increasing erythritol production in *Y. lipolytica*. We were further able to boost production by combining the expression of this enzyme with other approaches known to increase erythritol production in *Y. lipolytica*. This suggest that this new enzyme provides an orthogonal route for boosting production and can be stacked with existing designs known to increase sugar alcohol production in yeast such as *Y. lipolytica*. Collectively, this work establishes a new route for increasing sugar alcohol production and further develops *Y. lipolytica* as a promising host for erythritol production from cheap substrates such as glycerol.

**Supplementary Information:**

The online version contains supplementary material available at 10.1186/s13068-021-02039-0.

## Background

Erythritol is a four-carbon sugar alcohol used as a low-calorie sweetener in many foods, beverages, and chewing gums [1, 2]. In addition, erythritol can be chemically converted to platform chemicals such as 1,4-anhydroerythritol, butadiene, 1,4-butanediol, 2,5-dihydrofuran, and tetrahydrofuran [4, 5]. It is naturally found in fruits, seaweed, and alcoholic beverages at low concentrations [2, 3]. While erythritol can be chemically synthesized from 2-butene-1,4-diol, it is more commonly produced using yeast fermentations [2]. Examples of yeast capable of producing erythritol include *Moniliella*, *Pichia*, *Candida*, *Torula*, and *Yarrowia* [1, 6–17]. Among these yeasts, *Yarrowia lipolytica* is especially promising because it can produce erythritol from glycerol, a cheap byproduct of biodiesel production [14, 18, 19].

As a brief background, the microbial synthesis of erythritol from glycerol involves a multi-enzyme pathway [20, 21]. Glycerol is first phosphorylated by glycerol kinase (GK) and then subsequently dehydrogenated by glycerol 3-phosphate dehydrogenase (GPDH) to produce dihydroxyacetone phosphate. Triose phosphate isomerase next converts dihydroxyacetone phosphate into glyceraldehyde-3-phosphate, which then enters in pentose phosphate pathway. Transketolase (TKL) acts on glyceraldehyde 3-phosphate and sedoheptulose-7-phosphate to produce erythrose 4-phosphate and fructose-6-phosphate. Erythrose 4-phosphate is subsequently dephosphorylated by erythrose 4-phosphate phosphatase and then reduced by erythrose reductase (ER) to produce erythritol.

Erythritol is produced by many lactic acid bacteria, osmophilic yeasts, and fungi [1]. Among the microbes, osmophilic yeasts are arguably the most efficient organisms at producing erythritol. However, even then, further improvements in erythritol production are still possible. For example, UV and chemical mutagenesis was used to increase the erythritol yield by 40% in *Aureobasidium* sp. [22]. Similar approaches were also applied to *Moniliella* sp. and *Candida magnoliae* to increase erythritol production [13, 23].

Among the various osmophilic yeast capable of producing erythritol, *Yarrowia lipolytica* is particularly promising. This yeast is best known for its ability to produce citric acid and lipid-based chemicals at high titers [24–28]. It is also a GRAS organism with good genetic tools [29, 30]. In the context of this work, it can also produce erythritol from glucose or glycerol, though production is strain dependent [16, 31]. Indeed, there have been multiple reports focused on increasing erythritol production by optimizing the culture conditions using wild strains or randomly mutated ones [31–33].

In addition to production in native and randomly mutated strains, *Y. lipolytica* has also been genetically engineered to produce erythritol [16, 20, 33, 34]. In one study, GK and GPDH in *Y. lipolytica* were overexpressed. The resulting strain produced 1 g/L/h erythritol with a yield of 0.2 g/g from glycerol during fed-batch growth [33]. In another study, an erythrulose kinase deleted strain overexpressing glycerol kinase showed 74% higher productivity and 20% higher erythritol yields than the parental strain [20]. In a recent study of note, *Y. lipolytica* was metabolically engineered to produce erythritol from glucose [16]. The genes involved in the production of arabitol and mannitol and those involved in erythritol catabolism were deleted. The resulting strain produced erythritol with a yield of 0.65 g/g from glucose.

In this work, we investigated whether the heterologous expression of a sugar alcohol phosphatase (*PYP*), recently identified in *Saccharomyces cerevisiae* [35], would increase sugar alcohol production in *Y. lipolytica* PO1f, a derivative of *Y. lipolytica* W29 [36]. This *PYP* from *S. cerevisiae* prevents growth inhibition by sugar alcohol phosphates by hydrolyzing them to produce the corresponding sugar alcohols [35]. We first tested whether *Y. lipolytica* natively produces sugar alcohols during growth on different substrates with erythritol being the most promising. We also examined the effect of osmotic stress and found that it increased erythritol production during growth on glycerol. Consistent with these observations, osmotic stress was also found to increase the expression of genes involved in glycerol utilization. Analysis of intracellular metabolites revealed that amino acids, sugar alcohols, and polyamines were produced at higher levels from glycerol under osmotic stress. We next expressed the *S. cerevisiae PYP* in *Y. lipolytica* and found that it increased glycerol utilization and erythritol production. We were further able to increase erythritol production by increasing the expression of the native glycerol kinase and transketolase gene using both pure and crude glycerol as the substrate.

## Results

### *Yarrowia lipolytica* produces mannitol during growth on glucose, fructose, mannose, and glycerol in the nitrogen-rich medium

**Fig. 1:**
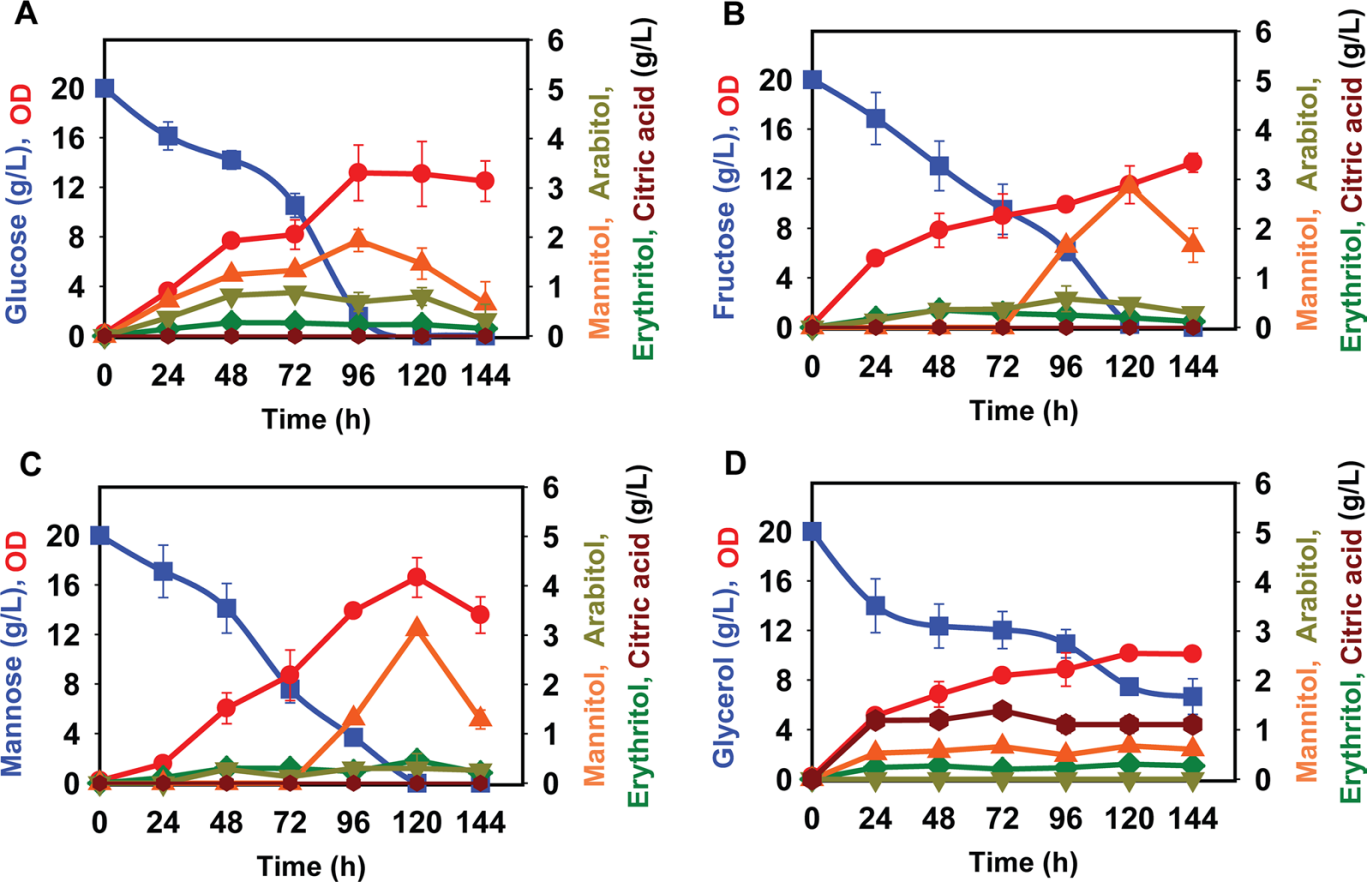
Growth of *Y. lipolytica* PO1f on different sugars at 20 g/L in yeast synthetic complete medium: **A** glucose, **B** fructose, **C** mannose, and **D** glycerol

*Yarrowia lipolytica* PO1f (hereafter referred to simply as *Y. lipolytica*) produces erythritol during growth on glycerol [14]. To determine whether *Y. lipolytica* produces any additional sugar alcohols during growth on carbon substrates other than glycerol, we examined growth on glucose, fructose, and mannose in nitrogen-rich medium. The cells were able to completely consume 20 g/L of glucose, fructose, and mannose after 144 h of shake-flask growth (Fig. 1 and Additional File 1: Fig. S1). We observed mannitol, arabitol, erythritol, and citric acid production during growth on these sugars using high-performance liquid chromatography (HPLC). In addition, these metabolites were identified by gas chromatography–mass spectrometry (GC–MS) and proton nuclear magnetic resonance spectroscopy (^1^H-NMR) (Additional File 2: Fig. S2, Additional File 3: Fig. S3, Additional File 4: Fig. S4, Additional File 5: Fig. S5, Additional File 6: Fig. S6). We next measured the concentration of mannitol, arabitol, erythritol, and citric acid during growth of *Y. lipolytica* (Fig. 1). Mannitol production from glucose peaked at concentrations of 2 ± 0.2 g/L after 96 h, while for fructose and mannose, it peaked at concentrations of 3 ± 0.1 g/L and 3.1 ± 0.2 g/L, respectively, after 120 h of growth. Arabitol and erythritol were produced at concentrations less than 0.5 g/L for all of the carbon sources studied. During growth on glycerol, less than 1 g/L of mannitol and erythritol were produced after 96 h of growth. In addition, we found that the glycerol was not completely consumed, most likely due to the drop in pH arising from citric acid production though the exact mechanism is unknown.

We also examined growth of *Y. lipolytica* on YP medium containing either glucose, fructose, mannose, or glycerol. The cells were able to completely consume 20 g/L of glucose, fructose, mannose, and glycerol after 48 h of shake-flask growth (Additional File 7: Fig. S7). We observed mannitol, arabitol, erythritol, and citric acid production during growth on these sugars. Erythritol production from fructose peaked at concentrations of 3.6 ± 0.1 g/L after 48 h, while for mannose and glycerol, it peaked at concentrations of 4.9 ± 0.1 g/L and 2.7 ± 0.2 g/L, respectively, after 48 h of growth. Citric acid was produced at concentrations of 2 to 4 ± 0.2 g/L after 48 h for all the carbon sources studied.

### Osmotic stress induces production of erythritol and arabitol in *Y. lipolytica*

**Fig. 2:**
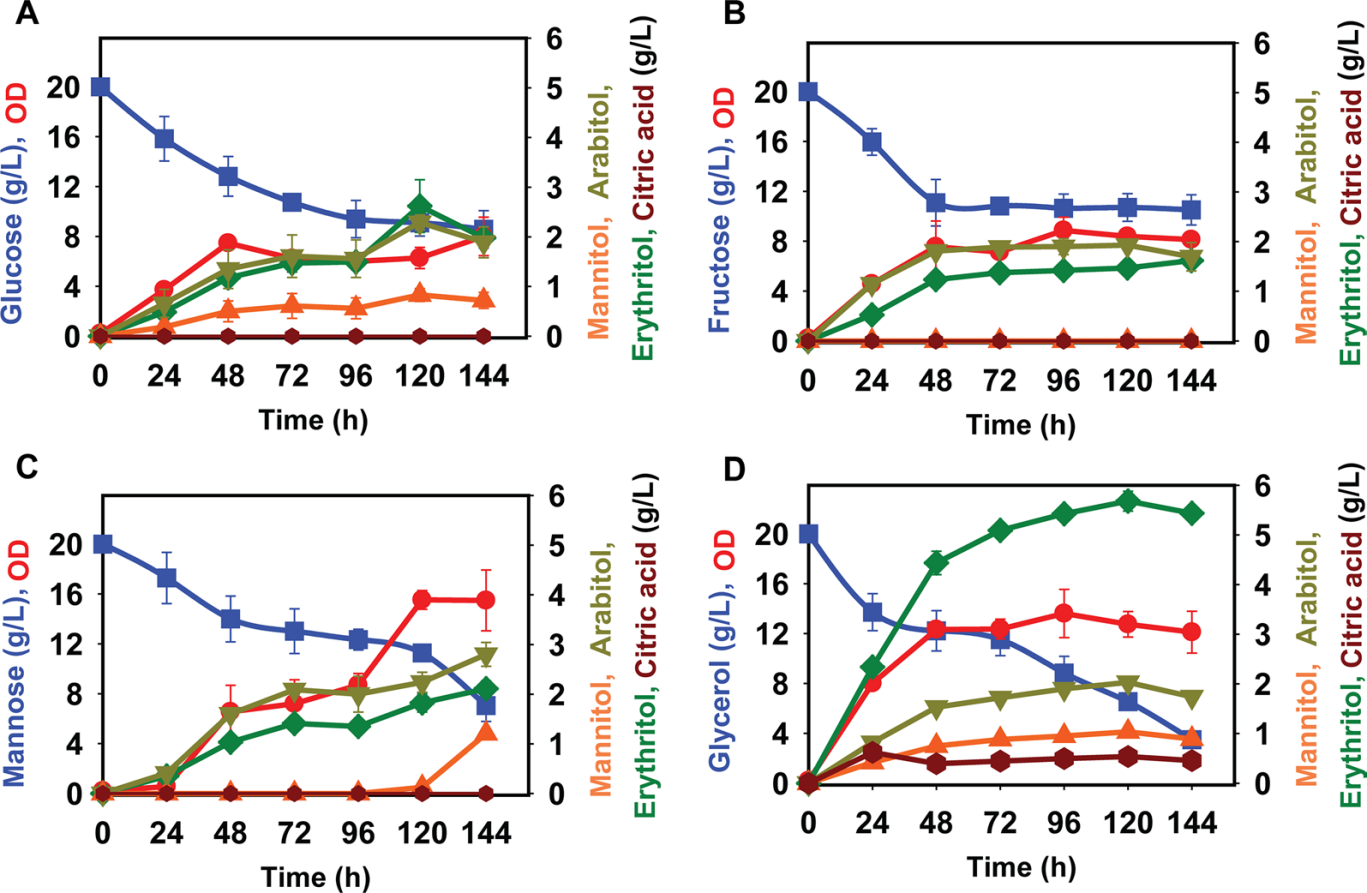
The effect of osmotic stress (NaCl) on sugar alcohol production in *Y. lipolytica* PO1f during growth on different sugars in yeast synthetic complete medium: **A** glucose, **B** fructose, **C** mannose, and **D** glycerol. Osmotic stress is introduced with the addition of 25 g/L of NaCl to YSC media

To determine the effect of osmotic stress on sugar alcohol production in *Y. lipolytica*, we analyzed growth on 20 g/L glucose, fructose, mannose, or glycerol with and without the addition of 2.5% NaCl in YSC medium. The addition of salt increased the production of arabitol and erythritol. Arabitol production from glucose, fructose and glycerol peaked at concentrations of 2 ± 0.2 g/L after 120 h, while production from mannose, peaked at concentrations of 2.8 ± 0.3 g/L after 144 h of growth (Fig. 2). The addition of salt strongly influenced erythritol production from glycerol. Erythritol production from glucose, fructose and mannose peaked at concentrations of 1.8 to 2 ± 0.2 g/L after 120 h. From glycerol, it peaked at a concentration of 5.8 ± 0.3 g/L after 120 h of growth. The addition of 2.5% NaCl led to incomplete utilization of glucose, fructose, and mannose, whereas the glycerol was almost utilized.

We also analyzed growth on YP medium containing 20 g/L glucose, fructose, mannose, or glycerol with the addition of 2.5% NaCl. The addition of salt increased the production of arabitol and erythritol and decreased the production of citric acid for all the carbon sources studied (Additional File 8: Fig. S8). These findings suggest that the osmotic stress response depends on the carbon source in *Y. lipolytica*. They also suggest that erythritol is the most promising sugar alcohol for production in *Y. lipolytica* and hence was the target for remainder of this work.

### A low C/N ratio increases erythritol production from glycerol

**Fig. 3:**
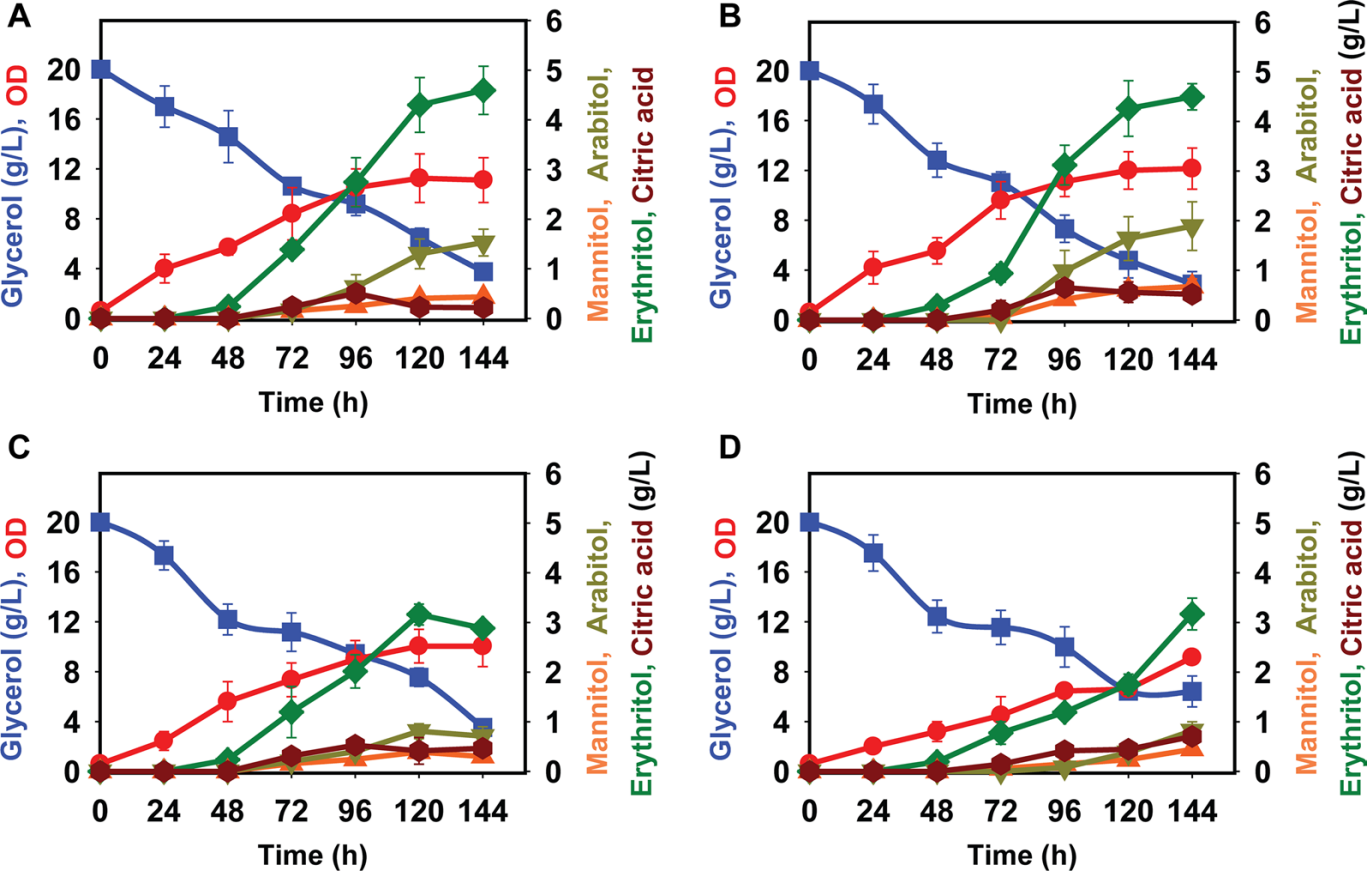
The effect of C/N ratio on erythritol production by *Y. lipolytica* PO1f using glycerol at 20 g/L in yeast minimal media: **A** C/N 5, **B** C/N 15, **C** C/N 30, and **D** C/N 60

Nitrogen starvation is a commonly used method to induce lipids and organic acid production from sugars in the oleaginous yeasts [34, 37, 38]. To determine the most suitable carbon-to-nitrogen (C/N) ratio for erythritol production from glycerol, we grew *Y. lipolytica* in minimal media containing 20 g/L glycerol at various C/N ratios ranging from 5 to 60. Erythritol was produced at all C/N ratios (Fig. 3). Among the C/N ratios tested, we found that a high C/N ratio (5 and 15) resulted in the highest production, with concentration exceeding 4.5 g/L erythritol after 144 h of growth.

We also analyzed the lipid content and composition in cells grown on 20 g/L glycerol in nitrogen-poor (C/N 60) and nitrogen-rich medium (C/N 5) after 144 h. In nitrogen-poor medium, the final dry cell weight was 3.3 g/L and the lipid content was 15%. In nitrogen-rich medium, the final dry cell weight was 4.9 g/L and lipid content was 5%. We also analyzed the composition of the lipid by fatty acid methyl ester analysis using GC–MS. The fatty acids were primarily oleic (C18:1) and palmitic (C16:0) acid, with some stearic (C18:0) acid in growth on nitrogen-poor and nitrogen-rich medium (Additional File 9: Fig. S9).

### Osmotic stress upregulated genes from pentose phosphate pathway and glycerol catabolic pathway

**Fig. 4:**
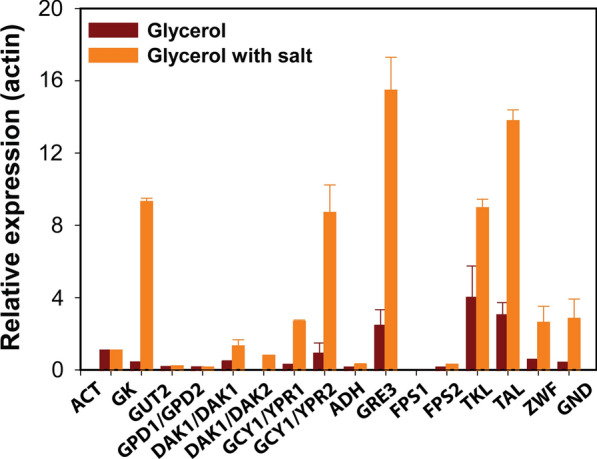
Expression profiles for genes during growth on glycerol (dark red color) and glycerol with salt (orange color) in nitrogen-rich medium. Genes are selected from the glycerol 3-P pathway, DHA pathway, glycerol uptake pathways, and PP pathway in *Y. lipolytica* PO1f. Gene expression levels were normalized based on the expression of the actin gene (*ACT*), glycerol kinase (*GK*), glycerol-3-P dehydrogenase (*GUT2*), glycerol-3-P dehydrogenase (*GPD1/GPD2*), dihydroxyacetone kinase (*DAK1/DAK1*), dihydroxyacetone kinase (*DAK1/DAK2*), glycerol dehydrogenase (*GCY1/YPR1*), glycerol dehydrogenase (GCY1/YPR2), arabitol dehydrogenase (ADH), aldose reductase (*GRE3*), aquaglyceroporin (*FPS1/FPS2*), transketolase (*TKL1*), transaldolase (*TAL1*), glucose-6-phosphate dehydrogenase (*ZWF1*), and 6-phosphogluconate dehydrogenase (*GND1*)

To better understand the mechanism governing osmotic stress-induced production of erythritol, we measured the expression of the genes involved in the glycerol uptake and metabolism using quantitative PCR during growth in YSC medium containing 2% glycerol with or without 2.5% NaCl (Fig. 4, Additional File 10: Table S1, Additional File 11: Table S2).

All of the genes were expressed during growth on glycerol alone or glycerol with salt. In the presence of salt, glycerol kinase (*GK*), dihydroxyacetone kinase (*DAK1*/*DAK2*), glycerol dehydrogenase (*GCY1*/*YPR1*), and aldose reductase (*GRE3*) showed the 32, 15, 10 and 4-fold higher expression as compared to the expression without osmotic stress. In addition, expression of transketolase (*TKL1*), transaldolase (*TAL1*), glucose-6-phosphate dehydrogenase (*ZWF1*), and 6-phosphogluconate dehydrogenase (*GND1)* were increased by 2- to 3-fold.

### Osmotic stress increases the production of sugar alcohols, polyamines, and amino acids

**Fig. 5:**
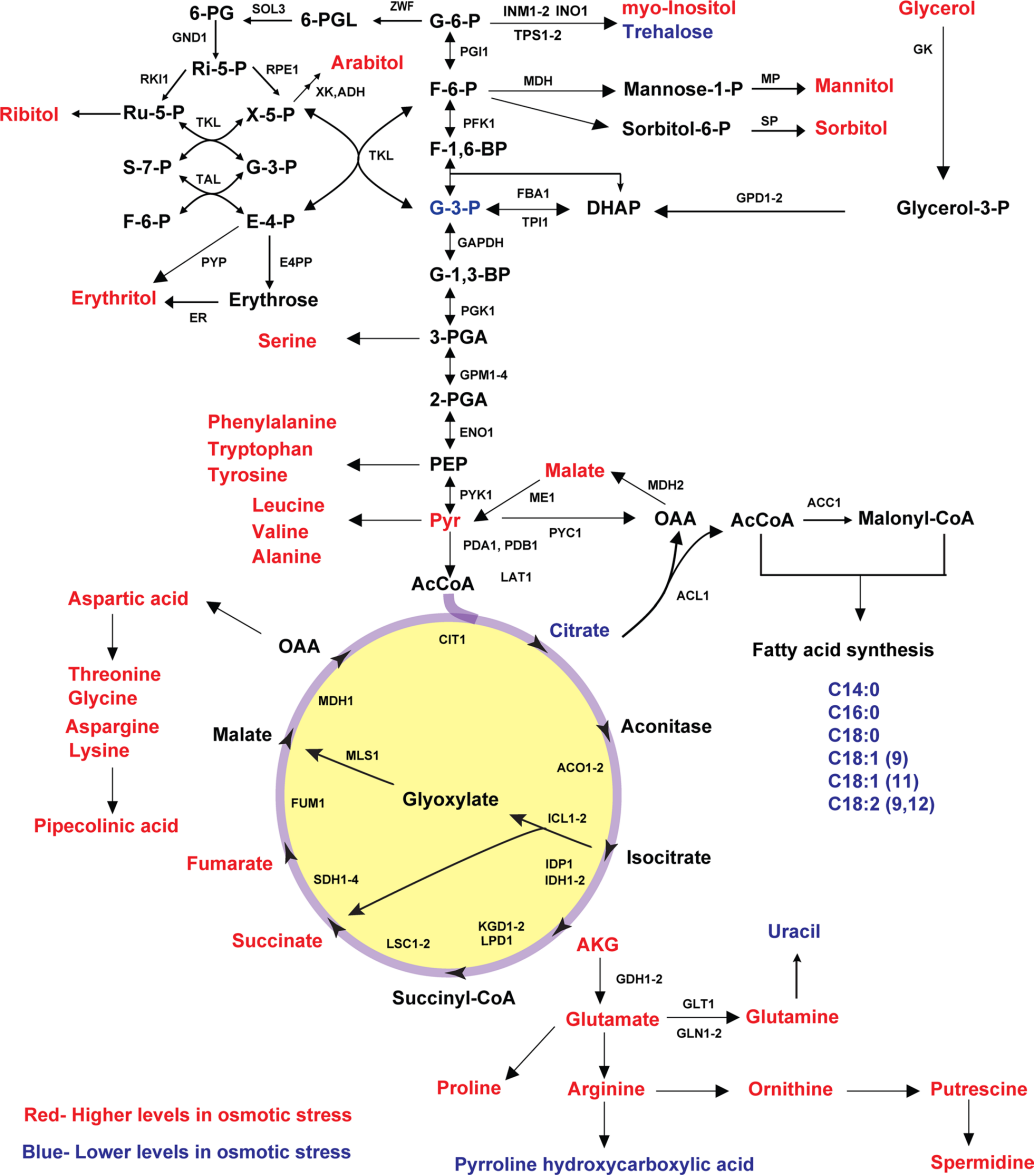
Changes in metabolite concentrations during growth of *Y. lipolytica* PO1f on glycerol versus glycerol with NaCl. Metabolites with relative higher concentration during growth on glycerol with NaCl as shown in red and metabolites with lower concentrations are shown in blue. 6-PGL: 6-phosphogluconolactonase, 6-PG: 6-phosphoglucolactone, Ru-5-P: ribulose-5-phosphate, X-5-P: xylulose-5-phosphate, Ri-5-P: ribose-5-phosphate, G-3-P: glyceraldehyde-3-phosphate, S-7-P: sedoheptulose 7-phosphate, E-4-P: erythrose 4-phosphate, G-6-P: glucose 6-phosphate, F-6-P: fructose 6-phosphate, F-1,6-BP: fructose 1,6-bisphosphate, G-3-P: glyceraldehyde 3-phosphate, DHAP: dihydroxyacetone phosphate, G-1,3-BP: glycerate 1,3-diphosphate, 3-PGA: 3-phospho-D-glycerate, 2-PGA: 2-phospho-D-glycerate, PEP: phosphoenolpyruvate, Pyr: pyruvate, AcCoA: acetyl CoA, AKG: alpha-ketoglutarate, OAA: oxaloacetate

We next compared the relative concentrations of 68 intracellular metabolites during exponential growth on glycerol containing 2.5% NaCl as compared to growth on glycerol alone using GC–MS (Fig. 5 and Additional File 12: Table S3). We have found many changes in the concentrations of amino acids, sugar alcohols, polyamines, and fatty acids. The concentration of 17 amino acids out of 20 amino acids was higher while growing on glycerol plus NaCl as compared to glycerol alone. Amino acids are a source of nitrogen and energy for the cell under stress conditions [40]. In particular, we observed increased concentrations of the glutamic acid (24×), proline (5.3×), phenylalanine (3.2×), aspartic acid (3×), glutamine (2.7×), leucine (2.2×), serine (2.1×), glycine (1.9×), and valine (1.3×) when salt was added. In the case of sugar alcohols, the addition of salt increased the intracellular concentration of arabitol (10.7×), erythritol (10.9×), ribitol (7.7×), inositol (3.2×), mannitol (2.6×), and sorbitol (1.07×), which fits their role in protecting cells against osmotic stress. Increased concentrations of pyruvate (1.3×), lactic acid (1.7×) and TCA intermediates such as α-ketoglutarate (1.8×), fumarate (1.5×), and succinate (1.2×) were also observed. We also observed an increase in polyamines such as putrescin (2.6×) and spermidine (41×) during growth in the presence of salt. These polyamines act as cell signaling molecules in enhancing tolerance to abiotic stresses and beneficial for protein homeostasis during abiotic stresses [41].

We also observed a decrease in the concentrations of the storage metabolite trehalose (0.28×), glycolytic intermediate glycerol 3-phosphate (0.76×), and glyoxylate cycle intermediate glyoxylic acid (0.48×) during growth in the presence of salt. Finally, we observed that salt decreased in the concentration of many fatty acids, C14:0 (0.6×), C16:0 (0.5×), C18:0 (0.6×), C18:1 (0.4×), C18:2 (0.8×), C20:0 (0.5×), and C22:0 (0.6×).

### Overexpression of sugar alcohol phosphatase and glycerol kinase increases glycerol consumption and erythritol production

*Saccharomyces cerevisiae* expresses a sugar alcohol phosphatase to prevent the inhibition of glycolysis by sugar alcohol phosphates. The phosphatase hydrolyzes sugar alcohol phosphates into corresponding sugar alcohols, and its expression is positively linked to growth [35]. The PYP is moderately active against erythrose-4-P, an intermediate in the erythritol biosynthesis pathway suggesting that it could increase erythritol production in *Y. lipolytica.* We specifically hypothesized that expression of a codon-optimized *PYP* in *Y. lipolytica* would result in increased erythritol production (Additional File 13: Table S4).

**Fig. 6:**
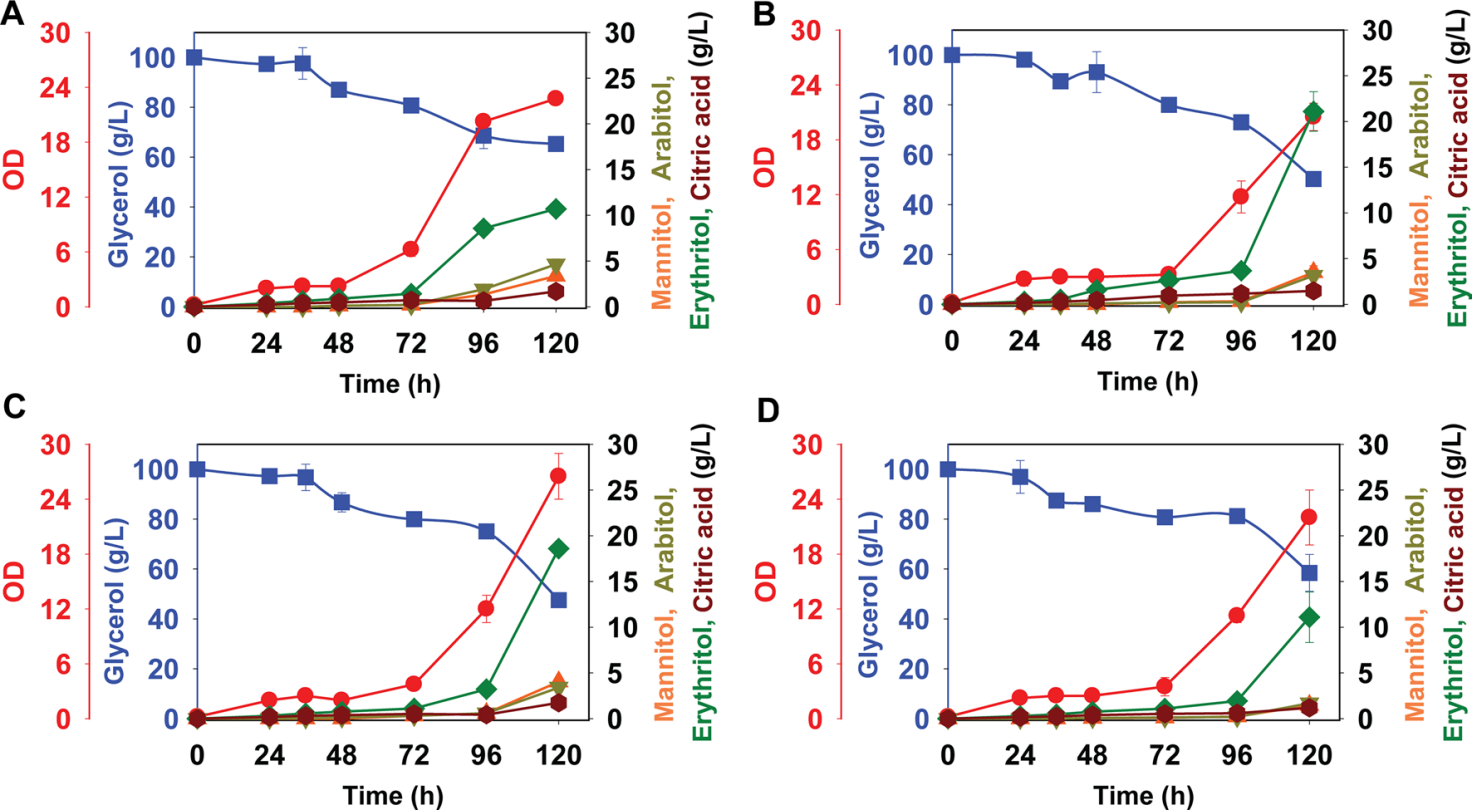
Production of erythritol by *Y. lipolytica* strains on glycerol (100 g/L) in PSM medium. PO1f (**A**), PO1f-*PYP* (**B**), PO1f-*GK* (**C**), and PO1f-*TKL* (**D**). Squares and circles were used to denote glycerol and OD, respectively, and plotted on left Y-axis. Triangles, inverted triangles, diamonds, and hexagons were used to denote mannitol, arabitol, erythritol, and citric acid, respectively, and plotted on right Y-axis

We expressed the codon-optimized *PYP* in *Y. lipolytica* and then tested the behavior of the resulting strain during growth in PSM medium containing 100 g/L glycerol. As shown in Fig. 6A, B, glycerol utilization and erythritol production were increased in the strain expressing the *PYP* relative to the PO1f control. The PYP expressing strain (PO1f-*PYP*) produced 18.6 ± 0.2 g/L erythritol following 120 h of growth on 100 g/L of glycerol, whereas the control strain (PO1f) produced 10.7 ± 0.2 g/L erythritol. In addition, the glycerol utilization was increased in the PO1f-*PYP* strain (49.8 ± 2.6 g/L versus 34.2 ± 2.2 g/L), though both it and the control were unable to consume all of the glycerol after 120 h of growth.

A number of studies have shown that increasing the expression of the native genes encoding for glycerol kinase and transketolase increases erythritol production in *Y. lipolytica* [16, 20, 33, 42]. We first tested whether increasing the expression of these gene would further increase erythritol production in PO1f control strain. PO1f-*GK* strain produced 21.1 ± 2.1 g/L erythritol (Fig. 6C) and the PO1f-*TKL* strain produced 11.1 ± 2.7 g/L erythritol (Fig. 6D). Glycerol utilization was increased in both strains (PO1f-*GK*: 52.4 ± 2.6 g/L and PO1f-*TKL*: 41.6 ± 7.3 g/L) though only the PO1f-*GK* strain produced more erythritol than the PO1f control. Fermentation parameters are provided in Table 1.

**Table 1:** The fermentation data for engineered strains versus wild type

Strain name	Erythritol (g/L)	Biomass (DCW g/L)	Time (h)	Qery (g/L h)	Yery (g/g glycerol)	R gly (g/L/h)	CP ery (g/g dcw h)
*Yarrowia lipolytica* O1f	10.70 ± 0.20	8.52	120	0.089	0.107	0.414	0.010
*Yarrowia lipolytica* PO1f-*PYP*P	18.60 ± 0.69	6.89	120	0.155	0.186	0.562	0.022
*Yarrowia lipolytica* PO1f-*GK*	21.09 ± 2.16	4.68	120	0.176	0.211	0.463	0.038
*Yarrowia lipolytica* PO1f-*TKL*	11.10 ± 2.76	4.68	120	0.092	0.111	0.471	0.020
*Yarrowia lipolytica* PO1f-*PYP*-*GK*	23.98 ± 0.15	12.74	120	0.200	0.240	0.792	0.016
*Yarrowia lipolytica* PO1f-*PYP*-*TKL*	15.26 ± 2.84	6.89	120	0.127	0.153	0.581	0.018
*Yarrowia lipolytica* PO1f-*PYP*-*GK*-*TKL*	27.48 ± 0.67	9.23	96	0.286	0.275	1.042	0.031

*Qery* productivity, *Yery* erythritol yield, *Rgly* glycerol consumption rate, *DCW* dry cell weight, *CPery* cell production rate

### The combined overexpression of sugar alcohol phosphatase, glycerol kinase, and transketolase further increases glycerol utilization rate and erythritol titers

**Fig. 7:**
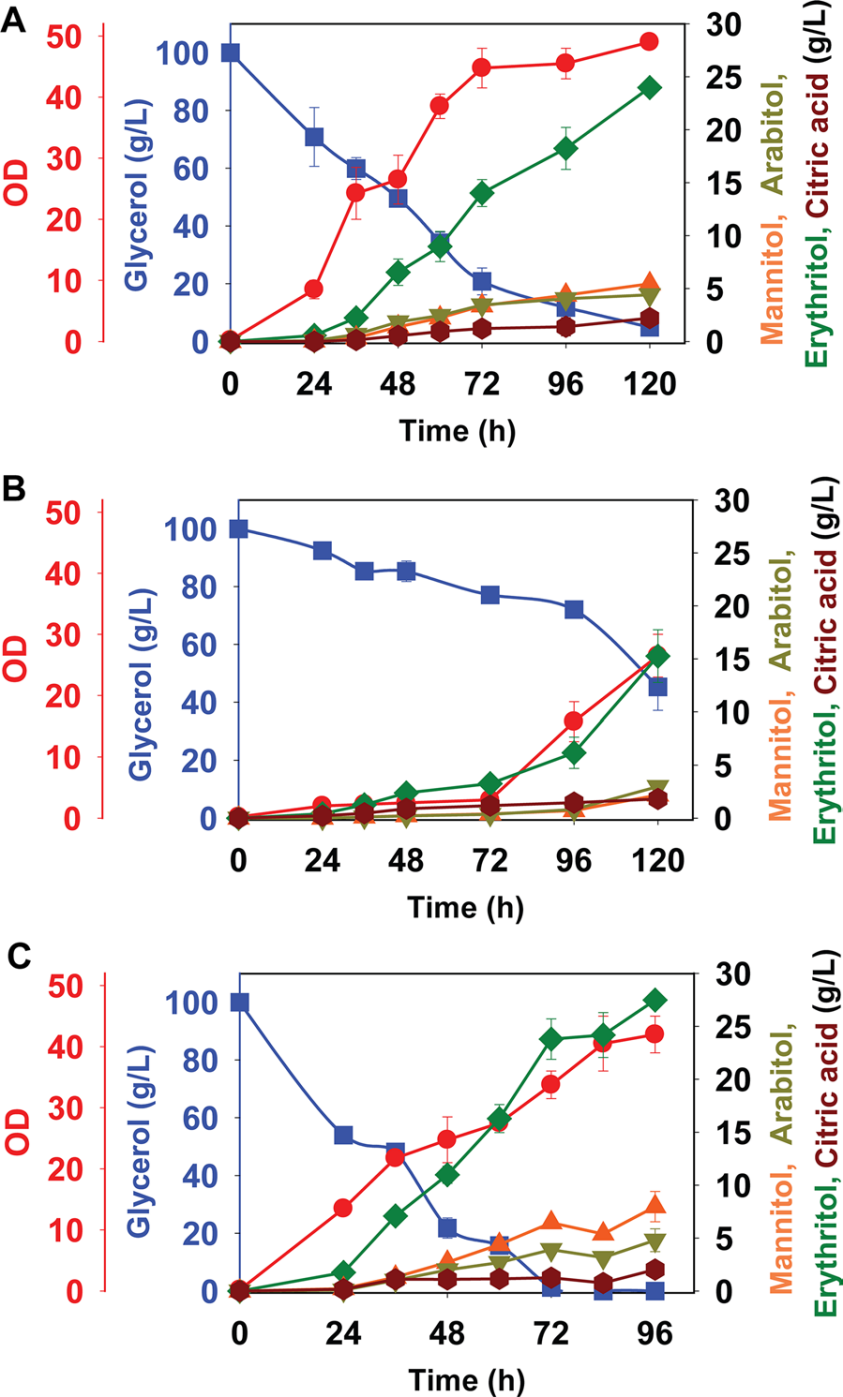
Production of erythritol by *Y. lipolytica* strains during growth on glycerol (100 g/L) in PSM medium. PO1f-*PYP*-*GK* (**A**), PO1f-*PYP*-*TKL* (**B**), and PO1f-*PYP*-*GK*-*TKL*. Squares and circles were used to denote glycerol and OD, respectively, and plotted on left Y-axis. Triangles, inverted triangles, diamonds, and hexagons were used to denote mannitol, arabitol, erythritol, and citric acid, respectively, and plotted on right Y-axis

**Fig. 8:**
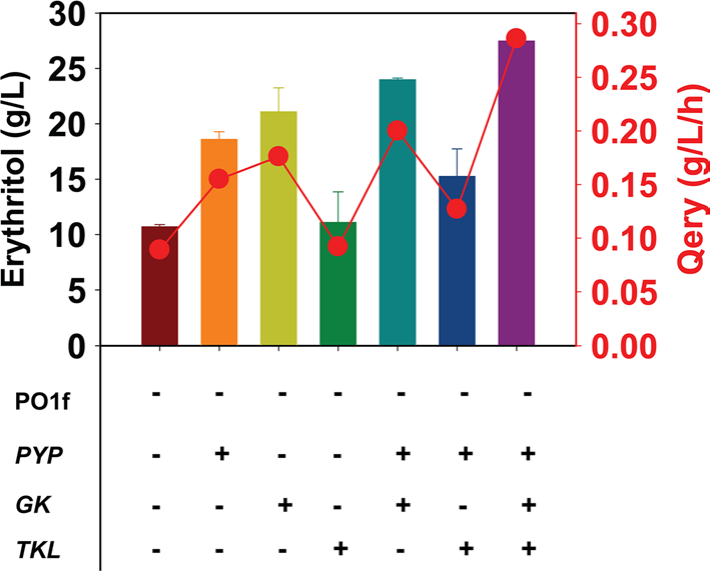
Erythritol titers and productivities for engineered strains of *Y. lipolytica* PO1f on glycerol (100 g/L) in PSM medium. Circles were used to denote glycerol utilization rate and plotted on left Y-axis

Our results demonstrate that expression of *PYP* and *GK* increased erythritol titers and glycerol utilization rates in *Y. lipolytica* PO1f though expression of *TKL* did not. We next tested whether strains expressing these genes in combination would further increase erythritol titer and glycerol utilization. We first transformed PO1f-*PYP* with expression cassettes for *GK*, *TKL*, or both, yielding the strains PO1f-*PYP*-*GK*, PO1f-*PYP*-*TKL*, and PO1f-*PYP*-*GK*-*TKL*. We then measured erythritol production during growth on 100 g/L glycerol. As shown in Fig. 7, erythritol titers were 23.9 ± 0.7 g/L, 15.2, ± 0.7 g/L, and 27.7 ± 0.7 g/L in the strains PO1f-P*YP*-*GK*, PO1f-*PYP*-*TKL*, and PO1f-*PYP*-*GK*-*TKL*, respectively. The highest erythritol productivity and erythritol yield were 0.29 g/L/h and 0.27 g/g glycerol for the PO1f-*PYP*-*GK*-*TKL* strain (Fig. 8). In addition, this strain was able to completely utilize all of the glycerol in the growth medium. The maximal glycerol utilization rate was 1.0 g/L/h for the PO1f-*PYP*-*GK*-*TKL* strain. The majority of erythritol was found in the spent medium. The intracellular concentration of erythritol in PO1f and PO1f-*PYP-GK-TKL* were 0.33 g/L (or 0.016 g/L/OD) and 4.74 g/L (or 0.117 g/L/OD) after 96 h, respectively (Additional File 14: Fig. S10).

### Erythritol production from crude glycerol

**Fig. 9:**
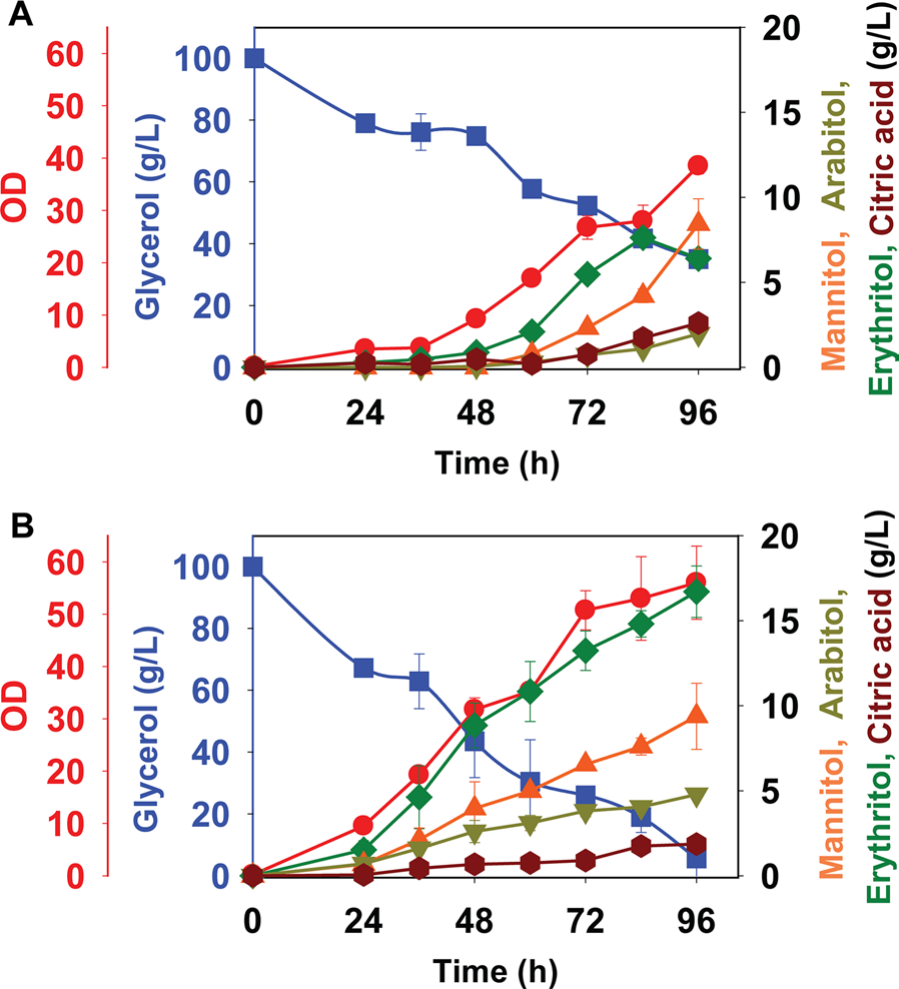
Production of erythritol by *Y. lipolytica* strains during growth on crude glycerol (100 g/L) in PSM medium. PO1f (**A**), and PO1f-*PYP*-*GK*-*TKL* (**B**). Squares and circles were used to denote glycerol and OD, respectively, and plotted on left Y-axis. Triangles, inverted triangles, diamonds, and hexagons were used to denote mannitol, arabitol, erythritol, and citric acid, respectively, and plotted on right Y-axis

We next examined erythritol production in PO1f and PO1f-*PYP*-*GK*-*TKL* using crude glycerol (100 g/L) obtained from a biodiesel production process in shake flasks. Erythritol titer and glycerol utilization rate for strain PO1f were 6.4 ± 0.6 g/L and 0.6 g/L/h after 96 h of growth (Fig. 9). Increased erythritol titers were observed 16.7 ± 1.5 g/L for the strain PO1f-*PYP*-*GK*-*TKL*.

### Erythritol overproduction from glycerol during fed-batch growth

**Fig. 10:**
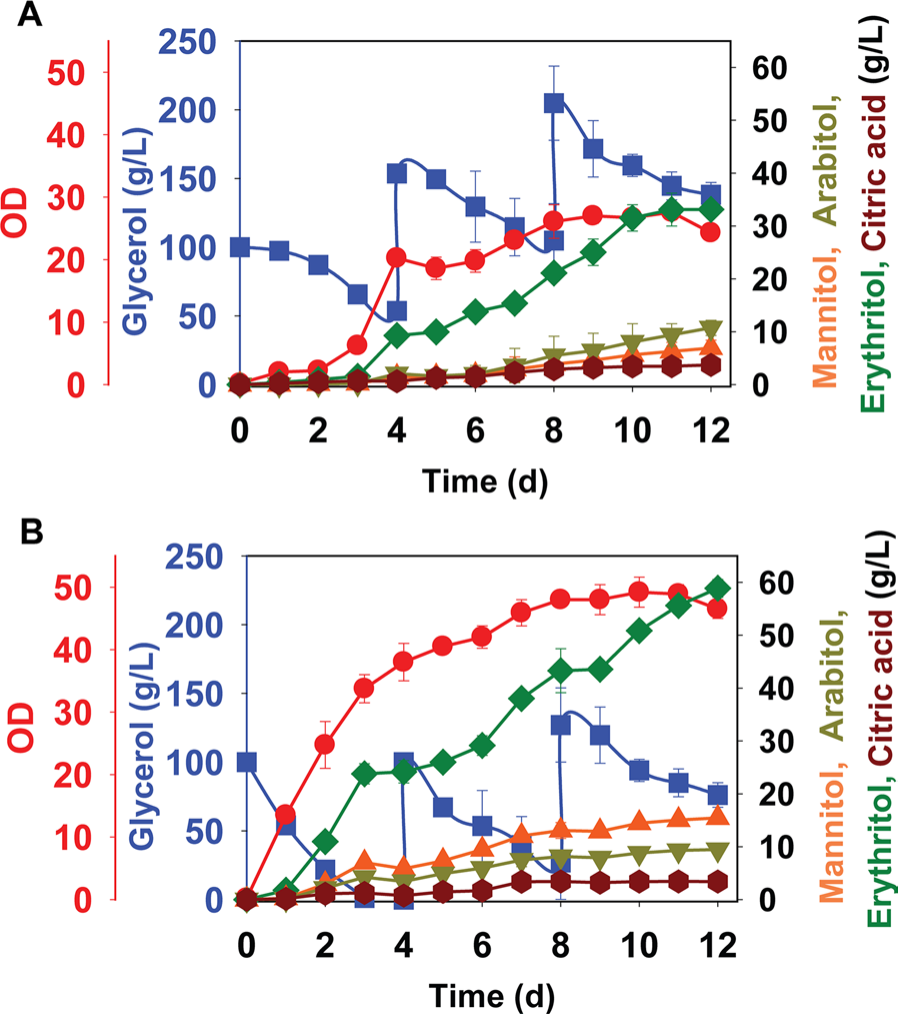
Growth profile and production of erythritol with additional metabolites for *Y. lipolytica* strains during fed-batch cultures on glycerol in PSM medium. PO1f (**A**), and PO1f-*PYP*-*GK*-*TKL* (**B**). Glycerol at 100 g/L was added in shake flasks after 4 days and 8 days reaching the total concentration of 300 g/L. Squares and circles were used to denote glycerol and OD, respectively, and plotted on left Y-axis. Triangles, inverted triangles, diamonds, and hexagons were used to denote mannitol, arabitol, erythritol, and citric acid, respectively, and plotted on right Y-axis

We next tested the performance of the PO1f-*PYP*-*GK*-*TK*L strain during fed-batch growth in shake flasks. The cells were grown with an initial, pure glycerol concentration of 100 g/L followed by additions of 100 g/L of pure glycerol every 4 days (equivalent to 25 g/L/day). Figure 10 shows the results over 12 days (288 h) of growth. The final erythritol titer for the PO1f-*PYP*-*GK*-*TKL* strain was 58.8 ± 1.7 g/L, an increase of 1.9-fold compared to PO1f. We also tested fed-batch growth using crude glycerol. However, we did not observe significant erythritol production in either the wild-type or engineered strain (data not shown), possibly due to the buildup of unknown contaminants within the crude glycerol.

## Discussion

In response to osmotic stress, *Y. lipolytica* produces erythritol and arabitol [33, 43]. Osmotic stress yields a significant increase in erythritol production during growing on glycerol. This sugar alcohol functions as an osmolyte that maintains the balance of cellular fluids [44]. The best example investigated is the accumulation of glycerol in *S. cerevisiae* in response to hyperosmolarity [45]. Oxidative stress is known to be an activator of the high-osmolarity glycerol (HOG) pathway in *C. albicans*, *S. pombe*, and *S. cerevisiae* [46]. The cellular response to oxidative stress in yeast is complex, involving MAPK signaling pathways [47–49]. A recent study investigated whether the erythritol production response to hyperosmotic stress is regulated by the HOG pathway in *Y. lipolytica* [43]. Deletion of the *HOG1* gene caused morphological defects, decreased a resistance to hyperosmotic stress, and negatively affected erythritol production. More research is required to determine whether this pathway plays a direct or indirect role in erythritol production as the molecular mechanism for inducing the erythritol production pathway associated with osmotic stress is still unclear.

To better understand changes in metabolism associated with osmotic stress in *Y. lipolytica*, we measured changes in transcription and metabolite concentrations using qPCR and GC/MS, respectively. The link between high osmolality and erythritol production during growth on glycerol is observed in *Y. lipolytica*. Addition of salt resulted in increased expression of several genes involved in the glycerol and erythritol metabolic pathways, which is consistent with increased production of erythritol. Glycerol kinase is a key enzyme for controlling glycerol levels inside the cells by phosphorylating it to glycerol-3-phosphate and subsequently incorporation into the pentose phosphate pathway to produce erythritol. Addition of salt upregulated glycerol kinase and pentose pathway genes to increase precursor levels for erythritol production. We also investigated changes in intracellular metabolites production in *Y. lipolytica* in response to addition of salt. The addition of salt has increased the biosynthesis of amino acids, sugar alcohols, and polyamines. This reflects an increased use of amino acids to produce energy and improve abiotic stress tolerance during growth on glycerol and NaCl.

To date, most studies to enhance erythritol production have used wild yeast strains or randomized mutagenized ones [1]. In addition, numerous studies have focused on optimizing the growing conditions and media optimizations to increase the erythritol production [14, 19, 31, 33]. These classic approaches often yield strains that are difficult to understand and further improve. Metabolic engineering, on the other hand, potentially provides an easier route for removing the bottlenecks within an existing pathway and for shifting metabolic flux towards the desired product. In this work, we explored a number of different metabolic engineering strategies for increasing erythritol production in *Y. lipolytica* during growth on glycerol.

A number of studies have shown that overexpression of the genes encoding for glycerol kinase and transketolase increase erythritol production in *Y. lipolytica* [16, 20, 33, 42]. We found that overexpressing the *GK* in *Y. lipolytica* strain increased the erythritol productivity and yield of erythritol by twofold. However, we did not observe increased erythritol expression when TKL was overexpressed. Aside from native genes, we also tested whether the expression of a recently characterized sugar alcohol phosphatase from in *S. cerevisiae* would increase production [35]. We found that overexpression of the *PYP* gene increased erythritol production and glycerol utilization rates in the *Y. lipolytica* strain. We further demonstrated that the co-expression of sugar alcohol phosphatase with glycerol kinase and transketolase further increases erythritol production and the rate of glycerol utilization in *Y. lipolytica* strain. Our best strain, PO1f-*PYP*-*GK*-*TKL*, produced erythritol at more than twice the level of the wild type during batch growth and fed-batch growth. This suggests that PYP can provide an orthogonal route for increasing erythritol production in *Y. lipolytica* and can potentially be combined with other published designs for increasing sugar alcohol production in yeast.

We also found that *Y. lipolytica* efficiently produces erythritol not only from pure glycerol, but also from crude glycerol. However, further work is necessary to identify the inhibitors within crude glycerol, which presumably limits growth during fed-batch fermentation. Biodiesel production generates approximately 10% (w/w) of glycerol as a major byproduct. The world biodiesel market has reached 2.8 billion gallons in the 2020, which indicated that approximately 280 million gallons of crude glycerol would be generated [50, 51]. The price of crude glycerol is $0.05 per pound. Therefore, development of sustainable processes using crude glycerol to produce value-added products is important to reduce the cost of biodiesel. Thus, application of crude glycerol in erythritol synthesis can reduce production costs.

We finally note that many *Y. lipolytica* strains natively produce erythritol far better than the PO1f strain used in this study. We focused on the PO1f stain because it has good genetic tools and much is known about its biology, mostly in the context of lipid and lipid-based chemical production. One potential concern is that PO1f strain is a uracil and leucine auxotroph [36]. One possibility is these auxotrophies may somehow limit production. To test this hypothesis, we constructed the corresponding prototroph (*URA* + , *LEU* +) and tested whether the resulting *PYP-GK-TKL* (*URA* + , *LEU* +) strain exhibited increased erythritol production. No significant differences were observed between auxotrophic and the prototrophic strains (data not shown). This is likely due to PSM medium not requiring leucine or uracil supplementation since it contains yeast extract.

## Conclusions

In this study, we demonstrated that arabitol and erythritol production in *Y. lipolytica* is associated with osmotic stress while growing on multiple sugars and glycerol. We were able to measure the transcription and metabolite changes associated with the osmotic stress in *Y. lipolytica* during growth on glycerol. We next metabolically engineered *Y. lipolytica* to increase erythritol production by exploring a number of different strategies. We also demonstrated the application of a promising enzyme sugar alcohol phosphatase for sugar alcohol production in *Y. lipolytica*, where erythritol production in *Y. lipolytica* can be increased by overexpressing this heterologous phosphatase. We were ultimately able to increase erythritol titers during batch growth and fed-batch growth by expressing the sugar alcohol phosphatase in combination with the native glycerol kinase and transketolase. Moreover, we obtained twofold higher erythritol titers when using crude glycerol as the substrate. Our results emphasize the potential of *Y. lipolytica* PO1f as a highly resourceful host for the sustainable production of erythritol.

## Methods

### Reagents

All chemicals were purchased from Sigma-Aldrich (St. Louis, MO) unless noted otherwise. All enzymes used for cloning and PCR were purchased from New England Biolabs (NEB, Ipswich, MA). Plasmid minipreps, PCR purifications, and gel extractions were done using the QIAprep Spin Miniprep Kit (Qiagen, Germantown, MD) and DNA Clean and Concentrator kit (Zymo Research, Irvine, CA), respectively. Genomic DNA from *Y. lipolytica* was extracted using the Quick-DNA Fungal/Bacterial Microprep Kit (Zymo Research, Irvine, CA). All oligonucleotides and gBlocks were purchased from Integrated DNA Technologies (Coralville, IA).

### Strains and growth medium

*Escherichia coli* 10-beta competent cells from New England Biolabs (Ipswich, MA) were used for cloning and propagation of plasmids in Luria–Bertani (LB) media supplemented with 100 μg mL^−1^ ampicillin. Plasmids and yeast strains used in this study are listed in Table 2. *Yarrowia lipolytica* PO1f (*MATa leu2-270 ura3-302 xpr2-322 axp1*) was obtained from the American Type Culture Collection (ATCC no. MYA-2613). It was used as the base strain for all experiments in this study. *Y. lipolytica* PO1f was maintained at 30 °C in YPD medium (1% yeast extract, 1% peptone, 2% glucose) or yeast synthetic complete (YSC) medium that contained 20 g/L glucose, 0.79 g/L complete supplement mixture (CSM) supplement, and 6.7 g/L yeast nitrogen base (YNB) from MP Biomedicals (Santa Ana, CA). CSM with the desired drop out (0.77 g/L CSM-URA, 0.69 g/L CSM-LEU and 0.67 g/L CSM-LEU-URA) was purchased from Sunrise Science Products (San Diego, CA). CSM-URA, CSM-LEU, CSM-LEU-URA, and YPD media with 150 ug/mL of hygromycin B were used for selecting transformants on agar plate.

**Table 2:** Plasmids and strains used in this study

	Description	Reference
Plasmids		
pHR_A08	UAS1B8-TEF-CYC1, *URA*, *AmpR*	This study
pINT3	UAS16B-CYCt, *LEU*, *AmpR*	This study
pCHPH	TEFintron-CYC1, *HYG*, *AmpR*	This study
pHR_A08_*PYP*	UAS1B8-TEF-*PYP*-CYC1, *URA, AmpR*	This study
pINT3_*GK*	UAS16B-*GK*-CYCt, *LEU,* A*mpR*	This study
pCHPH_*TKL*	TEFintron-*TKL*-CYC1, *HYG, AmpR*	This study
Strains		
* Escherichia coli-10 beta*	Δ(ara-leu) 7697 araD139 fhuA ΔlacX74 galK16 galE15 e14-ϕ80dlacZΔM15 recA1 relA1 endA1 nupG rpsL (StrR) rph spoT1 Δ(mrr-hsdRMS-mcrBC)	New England Biolabs
*Yarrowia lipolytica* PO1f	*MATa LEU2-270 URA3-302 xpr2-322 axp1*	ATCC MYA-2613
*Yarrowia lipolytica* PO1f-A08	*Y. lipolytica* PO1f integrated with linearized plasmid pHR_A08, *URA* +	This study
*Yarrowia lipolytica* PO1f- pINT3	*Y. lipolytica* PO1f integrated with linearized plasmid pINT3, *LEU* +	This study
*Yarrowia lipolytica* PO1f- pCHPH	*Y. lipolytica* PO1f integrated with linearized plasmid pCHPH, *HYG* +	This study
*Yarrowia lipolytica* PO1f-A08-pINT3	*Y. lipolytica* PO1f-A08 integrated with linearized plasmid pINT3, *URA* + *, LEU* +	This study
*Yarrowia lipolytica* PO1f-A08-pCHPH	*Y. lipolytica* PO1f-A08 integrated with linearized plasmid pCHPH, *URA* + *, HYG* +	This study
*Yarrowia lipolytica* PO1f-A08-pINT3-pCHPH	*Y. lipolytica* PO1f-A08-pINT3 integrated with linearized plasmid pCHPH, *URA* + *, LEU* + *, HYG* +	This study
*Yarrowia lipolytica* PO1f-*PYP*	*Y. lipolytica* PO1f integrated with linearized plasmid pHR_A08_*PYP*, *URA* +	This study
*Yarrowia lipolytica* PO1f-*GK*	*Y. lipolytica* PO1f A08 integrated with linearized plasmid pINT3_*GK*, *LEU* +	This study
*Yarrowia lipolytica* PO1f-*TKL*	*Y. lipolytica* PO1f pINT3 integrated with linearized plasmid pCHPH_*TKL*, *HYG* +	This study
*Yarrowia lipolytica* PO1f-*PYP-GK*	*Y. lipolytica* PO1f-*PYP* integrated with linearized plasmid pINT3_*GK*, *URA* + , *LEU* +	This study
*Yarrowia lipolytica* PO1f-*PYP-TKL*	*Y. lipolytica* PO1f-*PYP* integrated with linearized plasmid pCHPH_*TKL*, *URA* + , *HYG* +	This study
*Yarrowia lipolytica* PO1f-*PYP-GK-TKL*	*Y. lipolytica* PO1f-*PYP-GK* integrated with linearized plasmid pCHPH_*TKL*, *URA* + , *LEU* + *, HYG* +	This study

The following YSC media were tested for erythritol production (6.7 g/L yeast nitrogen base, 5 g/L ammonium sulfate, 0.79 g/L CSM supplement, and 20 g/L glucose, fructose, mannose or glycerol). Osmotic stress response was investigated in YSC media supplemented with 25 g/L of NaCl (6.7 g/L yeast nitrogen base, 5 g/L ammonium sulfate, 0.79 g/L CSM supplement, 25 g/L of NaCl, and 20 g/L glucose, fructose, mannose or glycerol). The effect of different C/N ratio on erythritol production media was investigated by cultivation in YSC glycerol medium with varying concentrations of ammonium sulfate (1.7 g/L yeast nitrogen base without amino acids and ammonium sulfate, 0.1–5 g/L (NH_4_)_2_SO_4_, 0.79 g/L CSM supplement, 25 g/L NaCl, 20 g/L glycerol, and 10 mM phosphate buffer). YPD medium, YPF medium (10 g/L yeast extract, 20 g/L peptone, and 20 g/L fructose), YPM medium (10 g/L yeast extract, 20 g/L peptone, and 20 g/L mannose), and YPG medium (10 g/L yeast extract, 20 g/L peptone, and 20 g/L glycerol) were tested for erythritol production. pH of YPD, YPF, YPM, and YPG media were set to 7.2 using 100 mM phosphate buffer. Osmotic stress response was investigated in YPD, YPF, YPM, and YPG media supplemented with 25 g/L of NaCl. Erythritol synthesis was also conducted in previously reported sugar alcohol synthesis medium (PSM medium) containing 100 g/L glycerol, 2.3 g/L (NH_4_)_2_SO_4_, 1 g/L MgSO_4_ × 7H_2_O, 0.23 g/L KH_2_PO_4_, 26.4 g/L NaCl, 1 g/L yeast extract, and 3 g/L CaCO_3_, pH 3.0. Following previous work [42], CaCO_3_ was added after the pH was adjusted to 3 in order to further acidification. In the fed-batch cultures, the initial PSM medium contained a pure glycerol concentration of 100 g/L. Additional glycerol was added at concentration of 100 g/L after 4 and 8 days of cultivation. Stationary phase seed cultures were obtained by inoculating single colonies from YPD agar plate into 20 mL YPD liquid medium in 125-mL glass baffled shake flasks. The seed cultures were then used to inoculate 50-mL cultures in 250-mL glass baffled shake flasks with a starting OD_600_ of 0.2. The cells were then grown at 30 °C and 250 rpm.

Crude glycerol was obtained from a 40 million gallons/year midwestern soy to biodiesel production facility that uses base-catalyzed transesterification of soy oil with methanol to produce biodiesel and crude glycerol. Briefly, the process consisted of a continuous transesterification reaction in a stirred tank reactor at 60 °C. Following a one-hour transesterification reaction, a continuous centrifuge was used to remove the glycerol-rich coproduct phase and processed through the glycerol recovery unit. It was composed (wt/wt) of glycerol 70–75%, NaCl 3–7%, and methanol less than 0.1%. Erythritol synthesis was conducted in PSM medium containing 100 g/L crude glycerol, 2.3 g/L (NH_4_)_2_SO_4_, 1 g/L MgSO_4_ × 7H_2_O, 0.23 g/L KH_2_PO_4_, 26.4 g/L NaCl, 1 g/L yeast extract, and 3 g/L CaCO_3_, pH 3.0. CaCO_3_ was added separately to each flask after establishing pH 3 to prevent a drop in pH. Citric acid production is enhanced at more neutral pHs in *Y. lipolytica* [14, 52]. Erythritol biosynthesis is also higher at low pH accompanied by low production of citric acid [14].

### Plasmid design and construction

The vectors and strains used in this study are listed in Table 2. The target genes and vector fragments were amplified with primer pairs from the templates listed in Additional File 11: Table S2 and Additional File 13: Table S4 and Additional File 15: Table S5. The gene encoding the sugar alcohol phosphatase (YNL010W) from *Saccharomyces cerevisiae* was codon-optimized and synthesized by Twist (GenBank: MW506864). Genomic DNA was isolated using the Quick-DNA Fungal/Bacterial Microprep Kit. The cell cultures at OD_600_ 10 were collected in centrifuge tubes and centrifuged at 6000 × g for 5 min at 4 °C. The supernatant was discarded and the pellet was used for genomic DNA extraction. The cells were resuspended in up to 200 µl of water or isotonic buffer to a ZR BashingBead lysis tube. The 750 µl BashingBead Buffer was added in the tube and homogenized using a FastPrep-24 homogenizer (MP Biomedicals, Irvine, CA, USA), beaten at a speed of 5 m/s for 30 s four times with cooling on ice between beatings. The cell lysates were purified according to the kit’s protocol. Native genes in the pentose phosphate pathway were obtained via PCR amplification of purified genomic DNA. *BssH*I and *Nhe*I used for cloning of *PYP* gene into pUAS1B8-TEF(136)-hrGFP. The genes encoding glycerol kinase (*GK*), and transketolase (*TKL*) were amplified from PO1f genomic DNA. *Hind*III and *Asc*I used for cloning of *GK* gene into pINT3 vector. The *TKL* gene is cloned into pCHPH vector. DNA fragments were ligated using ligation protocol with T4 DNA ligase. The *TKL* gene is cloned into pCHPH vector using Gibson assembly method [53]. Gel extractions were carried out using the Fermentas GeneJET Extraction Kit (Thermo Fisher Scientific, Waltham, MA). Plasmids were verified by digestion with restriction enzymes and by DNA sequencing.

### Transformation and strain engineering

Heterologous expression of the sugar alcohol phosphatase and overexpression of the pentose phosphate genes were carried out with linear DNA transformations using the different selectable markers. The vectors pHR_A08, pINT3, and pCHPH were linearized with SpeI, NotI, and BamHI-HF to obtain the control strains PO1f-A08 (*URA* +), PO1f-pINT3 (*LEU* +), and PO1f-pCHPH (*HYG* +), respectively (Table 2). Similarly, these vectors containing gene cassette of *PYP*, *GK*, and *TKL* were linearized with SpeI, NotI, and BamHI-HF to obtain the strains PO1f-*PYP* (*URA* +), PO1f-*GK* (*LEU* +), and PO1f-*TKL* (*HYG* +), respectively (Table 2). To obtain a control strain with different marker combinations, the linearized pINT3 (*LEU* +), pCHPH (*HYG* +) or combination of both (*LEU* + *, HYG* +*)* were transformed to PO1f-A08 (*URA* +) to obtain the strains PO1f A08-pINT3 (*URA* + *, LEU* +), PO1f-A08-pCHPH (*URA* + *, HYG* +), and PO1f-A08-pINT3-pCHPH (*URA* + *, LEU* + *, HYG* +), respectively. We also transformed PO1f-*PYP* with expression cassettes for GK, TKL, or both, yielding the strains PO1f-*PYP-GK* (*URA* + *, LEU* +), PO1f-*PYP-TKL* (*URA* + *, HYG* +), and PO1f-*PYP-GK-TKL* (*URA* + *, LEU* + *, HYG* +), respectively (Table 2). The linearized vectors with or without expression cassettes were column purified using the Zymo Research Clean and Concentrator kit. A transformation of *Y. lipolytica* PO1f with linearized plasmids with or without expression cassettes was performed according to modified protocol with selection on appropriate plates [34, 54]. Briefly, a single colony of *Y. lipolytica* PO1f was inoculated into 2 mL of YPD and incubated with shaking overnight at 30 °C. The overnight grown culture was diluted to OD 1 in 40 mL YPD and incubated at 30 °C for 5–6 h. Cells were harvested by centrifugation at 3000 × *g* for 3 min. The cells were then washed with sterile water, resuspended into a separate microcentrifuge tubes containing 100 mM LiOAc for transformation, and centrifuged again at 10,000 × *g* for 10 s. Cells were pelleted and LiOAc was removed using a micropipette. The transformation mix was added to the cells in following order: 90 ul 50% PEG4000, 5 ul 2 M LiOAc, 10 ul 1 M DTT, 12 ul 10 mg/mL of boiled salmon sperm DNA, and 0.5–2 ug linearized plasmid DNA. The microcentrifuge tube was vortexed until the cell pellet was completely mixed. Cells were incubated at 30 °C for 30 min and then heat shocked in a water bath at 42 °C for 30 min. Cells were pelleted to remove transformation mix and resuspended in 100 ul of sterile water. Cells were then plated on the appropriate selection agar plates. Transformation of linear DNA resulted in the integration of the fragment into a random location in the genome. Colonies were verified by PCR and then selected for erythritol production.

### Determination of sugar and sugar alcohol concentrations

Sugar, glycerol, and sugar alcohol concentrations were measured using a Shimadzu high-performance liquid chromatography system (Shimadzu, Columbia, MD) equipped with a RID-10A refractive index detector, an Aminex HPX-87H carbohydrate analysis column (Bio-Rad Laboratories, Hercules, CA), and a cation H micro-guard cartridge (Bio-Rad Laboratories) [55]. The column and guard cartridge were kept at 65 °C or 30 °C, and 5 mM H_2_SO_4_ was used as a mobile phase at a constant flow rate of 0.6 mL/min as previously reported. Prior to analysis, culture samples were first pelleted and then the supernatant was passed through 0.22-μm polyethersulfone syringe filter. Peaks were identified and quantified by retention time comparison to authentic standards.

Sugar and sugar alcohols peaks were also measured and confirmed by gas chromatography–mass spectrometry as previously reported (GC–MS) (Agilent Technologies, Santa Clara, CA) [55]. The 200 ul of sample was dried under vacuum and derivatized with 50 μL methoxyamine hydrochloride (40 mg/mL in pyridine) for 60 min at 50 °C, then with 150 μL of N-methyl-N-(trimethylsilyl)trifluoroacetamide plus 1% of trimethylchlorosilane at 70 °C for 2 h, and following 2 h incubation at room temperature. Chromatograms were acquired using a GC–MS system (Agilent) consisting of an Agilent 7890 gas chromatograph, an Agilent 5975 MSD and a HP 7683B autosampler. Gas chromatography was performed on a ZB-5MS (60 m × 0.32 mm I.D. and 0.25 mm film thickness) capillary column (Phenomenex, Torrance, CA). Inlet and MS interface temperatures were 230 °C, and the ion source temperature was adjusted to 230 °C. An aliquot of 1 mL was injected with a split ratio of 40:1. Helium carrier gas was kept at a constant flow rate of 2.4 mL/min. The temperature program was: the initial rate was held at 70 °C for 5 min followed by a ramp rate of 5 °C/min to 310 °C and final held for 10 min at 310 °C. The mass spectrometer was operated in a positive electron impact mode at 69.9 eV ionization energy at m/z 50–800 scan range. The spectra of all chromatogram peaks were evaluated using the AMDIS 2.71 (NIST) and authentic standards.

Sugar alcohols peak was also confirmed by proton nuclear magnetic resonance (^1^H-NMR) spectroscopy as previously reported [55]. Cells were grown for 96 h on 20 g/L glycerol in nitrogen-rich media and centrifuged at 16,000 × g for 10 min. The 600 μL of supernatant were mixed with 10% deuterium oxide. All 1D proton spectra were collected at 25 °C on an Agilent 750 MHz VNMRS spectrometer equipped with a 5-mm triple-resonance indirect-detection probe with Z PFG gradient capability [56]. The standard 1D presaturation pulse sequence were used, and water suppression was achieved with a low CW power irradiated at water resonance for 1.5 s. Each spectrum was collected with a 90° pulse angle of 10.5 ms, 32 scans and 2 s relaxation delay between scans. A solvent peak (H_2_O) was used as the chemical shift reference. Mnova 14.0.1 (Mestrelab Research) was used for spectral processing and analysis. Each spectrum was processed with 0.6 Hz line broadening, 4 times zero-filling, auto phase and auto baseline correction.

### Lipid and dry cell weight measurements

Lipids were also measured using the modified sulpho-phospho-vanillin lipid assay as previously reported [57–59]. The yeast culture was first centrifuged and then the cell pellet was washed twice using sterile water. The washed cell pellet was mixed with 1 mL of 18 M sulfuric acid in a glass test tube and heated at 100 °C for 10 min in a dry heating bath. The reaction was cooled for 20 min in an ambient water bath. The 2.5 mL of freshly prepared vanillin–phosphoric acid was added in the tubes and shaken for 15 min at 150 rpm at 37 °C in darkness. The test tube was cooled for 15 min in a water bath at ambient temperature. The absorbance of each reaction was measured at 530 nm against a reference sample prepared in water using the Tecan Infinite M1000 Pro microplate reader (Männedorf, Switzerland). Absorbance measurements were converted to lipid concentrations using a calibration curve prepared using refined corn oil. Corn oil (100 mg) was dissolved in 2:1 chloroform:methanol (20 mL) and a stock solution was loaded into an assay mixture at 50–250 µg. Corn oil solution was prepared freshly and a standard curve was run with each set. Vanillin–phosphoric acid solution was prepared freshly by dissolving 0.12 g vanillin in 20 mL dH_2_O and adjusting the volume to 100 mL with 85% o-phosphoric acid. This assay was validated previously for various oleaginous yeast lipids, and common media ingredients were found to provide minimal interference [57, 59].

Cell growth was measured by the optical absorbance at 600 nm (OD_600_). Dry cell weights (DCW) were determined as follows. Culture samples (1 mL) were collected into pre-weighed tubes and centrifuged at 16,000 × g for 5 min. Supernatant was discarded. Pellets were then washed twice with 50 mM phosphate buffered saline. Washed pellets were dried to constant weight at 65 °C for 24 to 48 h. The tubes were then weighed.

### Measurement of fatty acid methyl ester compositions

Fatty acid compositions were determined by GC–MS as previously reported [55]. The fatty acids were first derivatized using the method of Lepage and Roy [60]. Briefly, lyophilized samples (1 mL) were first resuspended in 2 mL of a 20:1 mixture of methanol and acetyl chloride solution and 2 mL of hexane. 25 mg/mL tridecanoic acid dissolved in a 3:2 methanol:benzene mixture was then added (2.5 μL) as an internal standard. The mixture was then incubated in a dry bath at 100 °C for 30 min in a sealed glass tube with a screw cap. After cooling down in room temperature, 1 mL water was added to induce phase separation. This process generates fatty acid methyl esters from all of the lipid compounds. The upper organic phase containing the fatty acid methyl esters was collected for analysis by GC/MS. Samples (2 μL) were injected at a 0:1 split ratio using hexane as a solvent. Helium carrier gas was used at a pressure of 121.7 kPa and a flow rate of 1.0 mL/min. The injection port temperature was set at 250 °C. Column temperature started at 30 °C and increased to 250 °C at a rate of 10 °C/min. Eluent from the GC entered an ionization chamber at 250 °C and was measured at a full scan between 15 and 250 amu. Species identity was verified by comparison of mass spectra to the analytical standards in the Sigma-Aldrich’s FAME mix (C8–C24) and NIST mass spectral library. Fatty acid methyl esters were quantified by peak area. Four replicates were analyzed for each sample (obtained from two biological replicates and two technical replicates).

### Quantitative PCR

Seed cultures at exponential phase were collected in the centrifuge tube and centrifuged at 6000 × g for 3 min at 4 °C. Supernatant was discarded and the pellets were resuspended in 1 mL of ddH_2_O. Seed cultures were then used to inoculate in nitrogen-rich glycerol medium with or without 25 g/L NaCl in a 125-mL baffled shake flask with a starting OD_600_ of 0.2 and incubated at 30 °C and 250 rpm. Growth experiments are performed with three biological replicates. Samples were collected after 24 h incubation at exponential phase. The cell cultures of total OD ~ 20 were collected in centrifuge tubes and centrifuged at 6000 × g for 3 min at 4 °C. Supernatant was discarded and pellet was used for the RNA extraction. Total mRNA was then extracted using Qiagen’s RNeasy Mini kit as previously described with a slight modification [52, 55]. *Y. lipolytica* Po1f cells (OD ~ 20) pellet was resuspended in 350 μL of Buffer RLT from the RNeasy mini kit. Approximately 500 μL of acid-washed glass beads (Sigma, acid washed, 425–600 μm) were added and homogenized using a FastPrep-24 homogenizer, beaten at a speed of 5 m/s for 30 s six times with cooling on ice between beatings. The cell lysates were purified according to the kit’s protocol purification of total RNA from yeast. Total mRNA (1 µg) was then treated with TURBO DNA-free DNase using the Ambion’s TURBO DNA-free kit (Thermo Fisher, Carlsbad, CA) to remove genomic DNA. cDNA was synthesized from mRNA using the Bio-Rad’s iScript cDNA synthesis kit. The qPCR experiments were carried out in 384 well plate using a Roche LightCycler 480 system with the SsoAdvanced Universal SYBR Green Supermix kit (Bio-Rad, Hercules, CA). Primers were designed using the online PrimerQuest tool provided by Integrated DNA Technologies and are listed in Additional File 8: Table S1. The actin gene (*ACT*) was used as an internal control. All data points were collected from three biological replicates.

### Analysis of intracellular metabolites

For intracellular metabolite analysis, a fast filtration sampling method was used as previously described with a slight modification [61, 62]. Briefly, 0.5 mL of culture grown until the exponential phase in YSC glycerol medium with or without 25 g/L NaCl was collected and vacuum-filtered using a vacuum manifold system (Vac-Man Laboratory Vacuum Manifold, Promega, Madison, WI) assembled with a nylon membrane filter (0.45 µm pore size, 13 mm diameter, Whatman, Piscataway, NJ) and a filter holder (Millipore). The filtered cell culture was then washed with 2.5 mL of distilled water at room temperature. The entire process for fast filtration was finished within 1 min. The filter membrane containing the washed cells was quickly mixed with 1 mL of the pre-chilled isopropanol:acetonitrile:water mixture (3:3:2, v/v). Cell samples were subsequently extracted by ultrasound (5 × 1 min) with QSonica Microson XL2000 Ultrasonic Homogenizer (Qsinica, LLC., CT) at 4 °C, centrifuged at 15,000 × g for 3 min 4 °C, and supernatants were collected and evaporated under vacuum.

Prior to GC/MS analysis, metabolome samples were derivatized with 100 μL methoxyamine hydrochloride (40 mg mL^-1^ in pyridine) for 90 min at 50 °C and then with 100 μL MSTFA at 50 °C for 120 min. Twenty microliters (20 μL) of the internal standard (hentriacontanoic acid, 1 mg/mL) was added to each sample prior to derivatization. Samples were analyzed on a GC/MS system (Agilent Inc., Palo Alto, CA) consisting of an Agilent 7890 gas chromatograph, an Agilent 5975 mass selective detector, and a HP 7683B autosampler. Gas chromatography was performed on a ZB-5MS (60 m × 0.32 mm I.D. and 0.25 um film thickness) capillary column (Phenomenex, CA). The inlet and MS interface temperatures were 25 °C, and the ion source temperature was adjusted to 230 °C. An aliquot of 1 uL was injected with the split ratio of 10:1. The helium carrier gas was kept at a constant flow rate of 2.4 mL/min. The temperature program was: 5-min isothermal heating at 70 °C, followed by an oven temperature increase of 5 °C/min to 310 °C and a final 10 min at 310 °C. The mass spectrometer was operated in positive electron impact mode (EI) at 69.9 eV ionization energy in m/z 30–800 scan range.

The spectra of all chromatogram peaks were evaluated using the AMDIS 2.71 (NIST, MD) software using a custom-built MSdatabase [63]. All known artificial peaks were identified and removed prior data mining. To allow comparison between samples, all data were normalized to the internal standard in each chromatogram and the cells dry weight (DW). The instrument variability was within the standard acceptance limit (5%).

## Supplementary Information

**Additional file 1:Figure S1.** Metabolic pathways in Yarrowia lipolytica PO1f to produce sugar alcohols and citric acid using multiple sugars and glycerol. Text box denotes the major secreted products during growth of Yarrowia lipolytica PO1f on sugars and glycerol.

**Additional file 2:Figure S2.** Gas chromatography-mass spectrometry analysis of sample peaks. (a) Gas chromatogram showing peaks for erythritol, arabitol, mannitol, and glucose, (b) Gas chromatogram showing peaks for erythritol, arabitol, mannitol, and fructose.

**Additional file 3:Figure S3.** Gas chromatography-mass spectrometry analysis of sample peaks. (a) Gas chromatogram showing peaks for erythritol, arabitol, mannitol, and mannose, (b) Gas chromatogram showing peaks for erythritol, arabitol, mannitol, citric acid, and glycerol.

**Additional file 4:Figure S4.** Gas chromatography-mass spectrometry analysis of sample peaks. Extracted mass spectra for glycerol, arabitol, erythritol, and citric acid.

**Additional file 5:Figure S5.** Gas chromatography-mass spectrometry analysis of sample peaks. Extracted mass spectra for fructose, glucose, mannose, and mannitol.

**Additional file 6:Figure S6.** Proton nuclear magnetic resonance (1H-NMR) spectroscopy analysis of sample peaks. A total of 6 spectra are shown in Figure (all samples were dissolved in 90% H2O and 10% D2O). Panel (a) contains the 1H spectrum of the substrate glycerol. Panel (b) shows the 1H spectrum of the culture media only. No erythritol and additional metabolite signals were detected. Panel (c) contains the 1H spectrum of the products from glycerol. The signals from erythritol show up clearly in this spectrum. Panel (d) is the spectrum collected after a few mg of erythritol powder was added directly to NMR tube. The signals from erythritol increased significantly, again indicating that the peaks in (d) are from erythritol. Panel (e) is the spectrum collected after a few mg of mannitol powder was added directly to NMR tube. Panel (f) is the spectrum collected after a few mg of arabitol powder were added directly to NMR tube.

**Additional file 7:Figure S7.** Growth of Y. lipolytica PO1f on different sugars at 20 g/L in YP medium: (A) glucose, (B) fructose, (C) mannose, and (D) glycerol.

**Additional file 8:Figure S8.** The effect of osmotic stress (NaCl) on sugar alcohol production in Y. lipolytica PO1f during growth on different sugars in YP medium: (A) glucose, (B) fructose, (C) mannose, and (D) glycerol. Osmotic stress was introduced with the addition of 25 g/L NaCl.

**Additional file 9:Figure S9.** Fatty acid composition as determined by GC–MS. Data show the mean and standard deviation resulting from two biological and two technical replicates.

**Additional file 10:Tables S1.** List of genes selected for quantitative PCR.

**Additional file 11:Table S2.** Oligonucleotides used in this study for qPCR.

**Additional file 12:Table S3.** A total of 68 intracellular metabolites such as amino acids, sugars, phosphates, fatty acids, organic acids, nucleosides and others, identified under glycerol without NaCl and glycerol with NaCl conditions by GC–MS analysis in Y. lipolytica Po1f.

**Additional file 13:Table S4.** Nucleotide sequence of codon optimized sugar alcohol phosphatase.

**Additional file 14:Figure S10.** The intracellular accumulation of erythritol in Yarrowia lipolytica PO1f and Yarrowia lipolytica PO1f-PYP-GK-TKL on glycerol (100 g/L) in PSM medium.

**Additional file 15:Table S5.** Oligonucleotides used in this study.

## Data Availability

The datasets used and/or analyzed during the current study are available from the corresponding author on reasonable request.

## Abbreviations

*PYP*: Sugar alcohol phosphatase
*GK*: Glycerol kinase
*TKL*: Transketolase
*ER*: Erythrose reductase
*GPDH*: Glycerol 3-phosphate dehydrogenase
*DAK*: Dihydroxyacetone kinase
*GCY*: Glycerol dehydrogenase
*GRE3*: Aldose reductase
*TAL*: Transaldolase
*ZWF*: Glucose-6-phosphate dehydrogenase
*GND*: 6-Phosphogluconate dehydrogenase
PSM: Sugar alcohol synthesis medium
HPLC: High-performance liquid chromatography
GC–MS: Gas chromatography–mass spectrometry
^1^H-NMR: Proton nuclear magnetic resonance spectroscopy
TCA: Citric acid cycle
YP: Yeast extract and peptone
YPD: Yeast extract, peptone and glucose
DCW: Dry cell weights
ACT: Actin gene
LB: Luria–Bertani medium
HOG: High-osmolarity glycerol
YSC: Yeast synthetic complete medium
CSM: Complete supplement mixture
*6-PGL*: 6-Phosphogluconolactonase
6-PG: 6-Phosphoglucolactone
Ru-5-P: Ribulose-5-phosphate
X-5-P: Xylulose-5-phosphate
Ri-5-P: Ribose-5-phosphate
G-3-P: Glyceraldehyde-3-phosphate
S-7-P: Sedoheptulose 7-phosphate
E-4-P: Erythrose 4-phosphate,
G-6-P: Glucose 6-phosphate,
F-6-P: Fructose 6-phosphate
F-1,6-BP: Fructose 1,6-bisphosphate
G-3-P: Glyceraldehyde 3-phosphate,
DHAP: Dihydroxyacetone phosphate
G-1,3-BP: Glycerate 1,3-diphosphate
3-PGA: 3-Phospho-D-glycerate
2-PGA: 2-Phospho-D-glycerate,
PEP: Phosphoenolpyruvate
Pyr: Pyruvate
AcCoA: Acetyl CoA
AKG: Alpha-ketoglutarate
OAA: Oxaloacetate
