# Supplementary figures and images for: Metabolic engineering of the oleaginous yeast Yarrowia lipolytica PO1f for production of erythritol from glycerol
Source: Biotechnol Biofuels. 2021 Sep 25;14:188. doi: 10.1186/s13068-021-02039-0 (PMC8466642; doi:10.1186/s13068-021-02039-0)

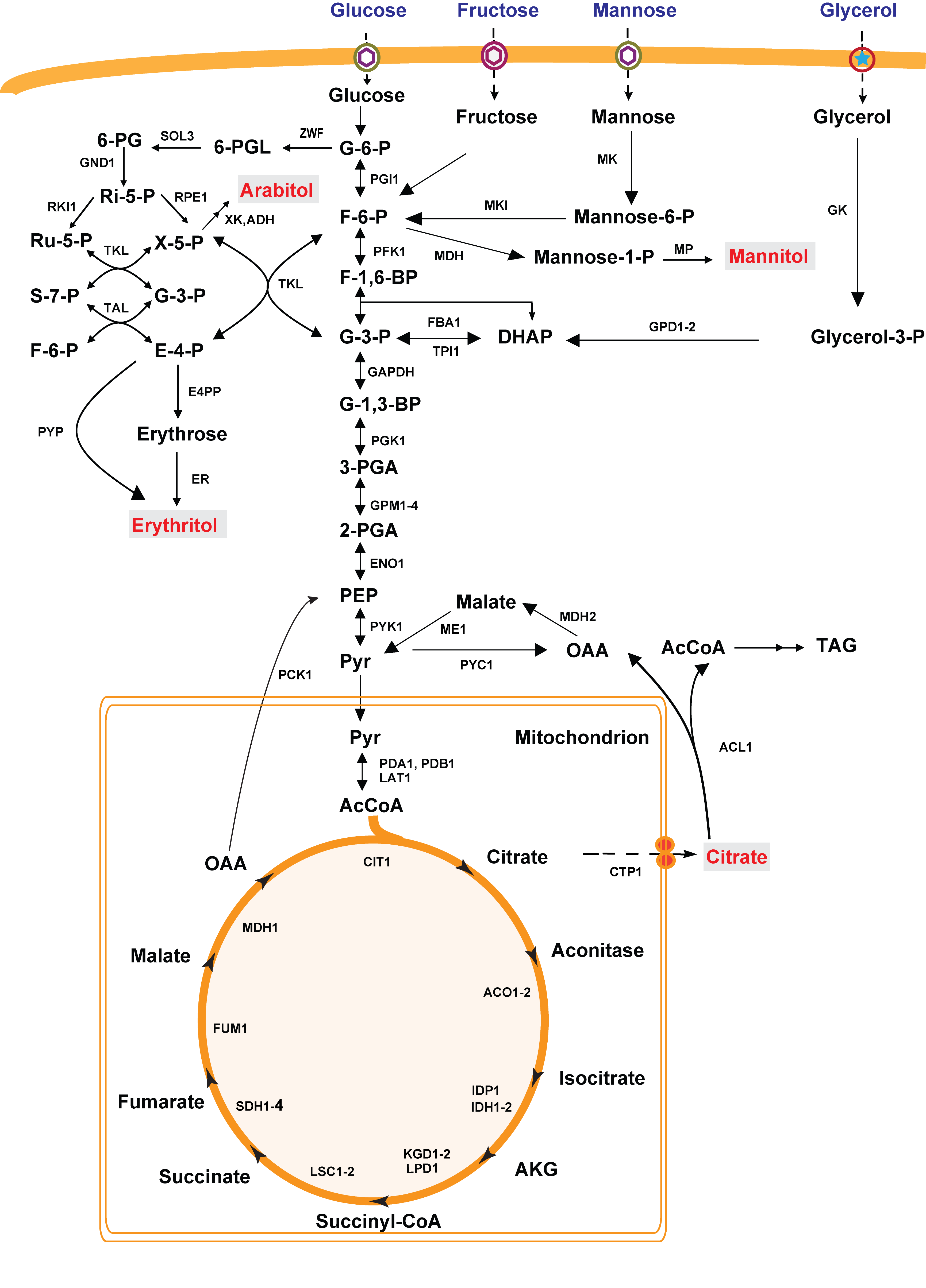

Supplement: Supplementary file 1 — Additional file 1:Figure S1. Metabolic pathways in Yarrowia lipolytica PO1f to produce sugar alcohols and citric acid using multiple sugars and glycerol. Text box denotes the major secreted products during growth of Yarrowia lipolytica PO1f on sugars and glycerol. [file 13068_2021_2039_MOESM1_ESM.tif]

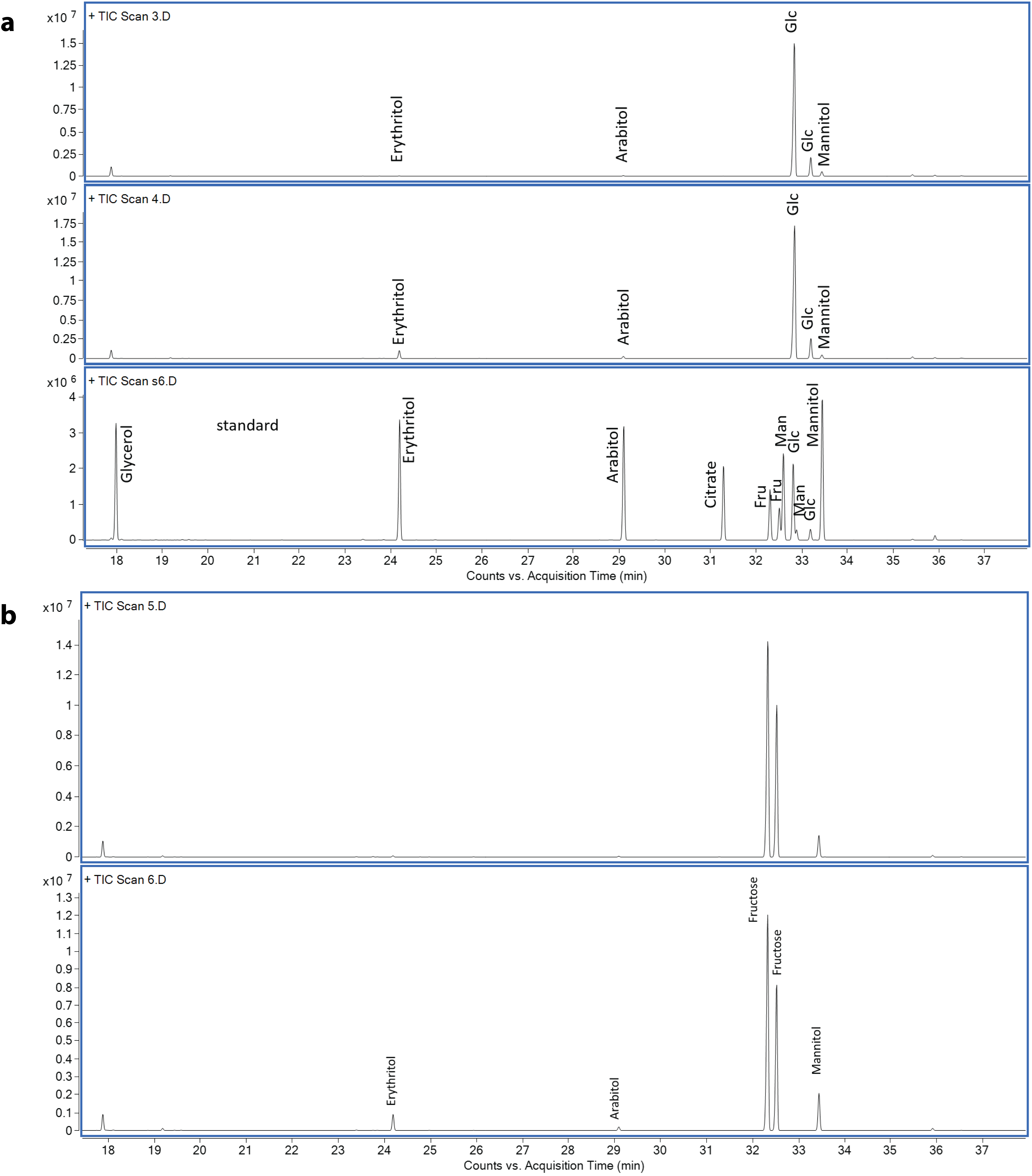

Supplement: Supplementary file 2 — Additional file 2:Figure S2. Gas chromatography-mass spectrometry analysis of sample peaks. (a) Gas chromatogram showing peaks for erythritol, arabitol, mannitol, and glucose, (b) Gas chromatogram showing peaks for erythritol, arabitol, mannitol, and fructose. [file 13068_2021_2039_MOESM2_ESM.tif]

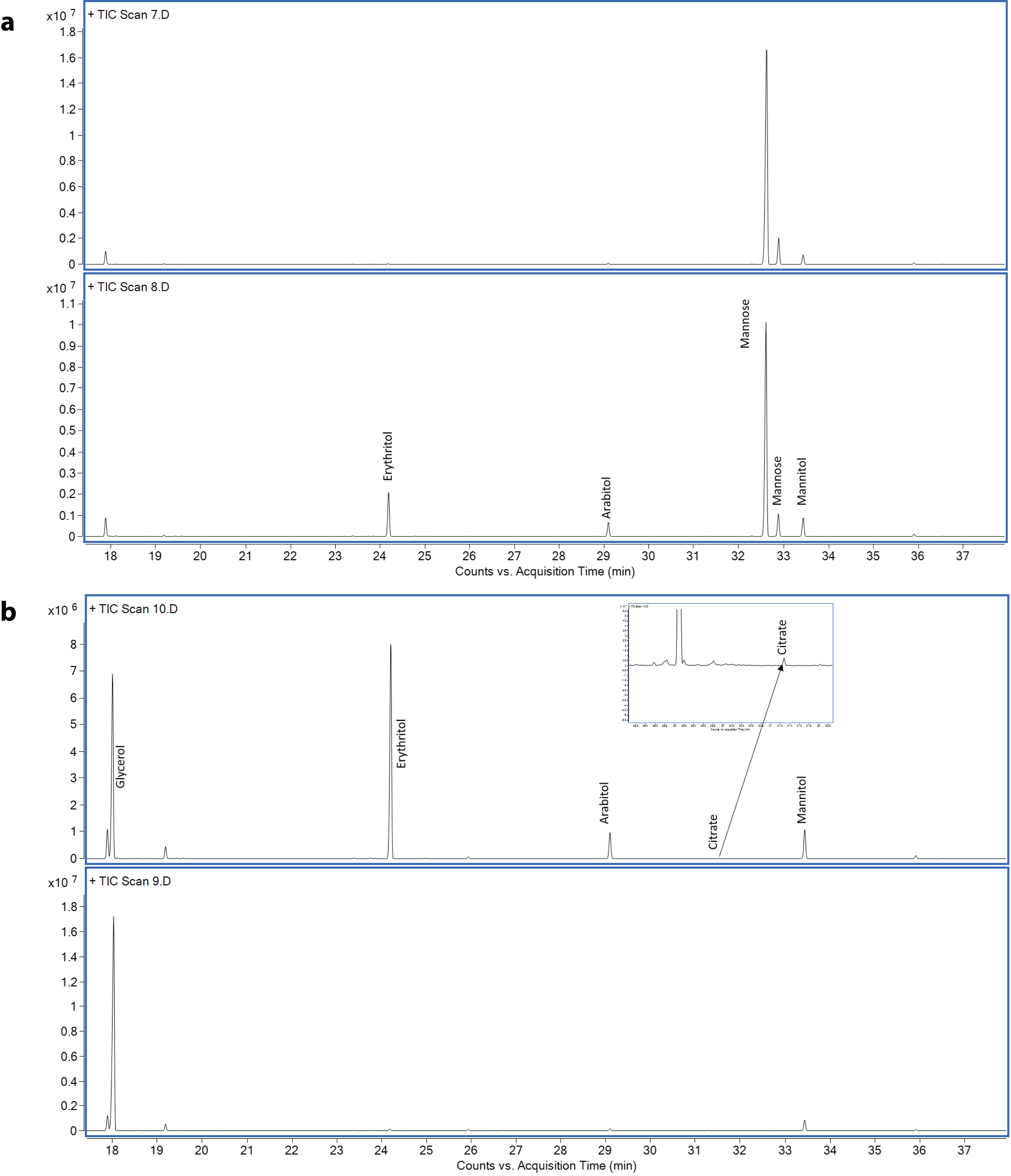

Supplement: Supplementary file 3 — Additional file 3:Figure S3. Gas chromatography-mass spectrometry analysis of sample peaks. (a) Gas chromatogram showing peaks for erythritol, arabitol, mannitol, and mannose, (b) Gas chromatogram showing peaks for erythritol, arabitol, mannitol, citric acid, and glycerol. [file 13068_2021_2039_MOESM3_ESM.tif]

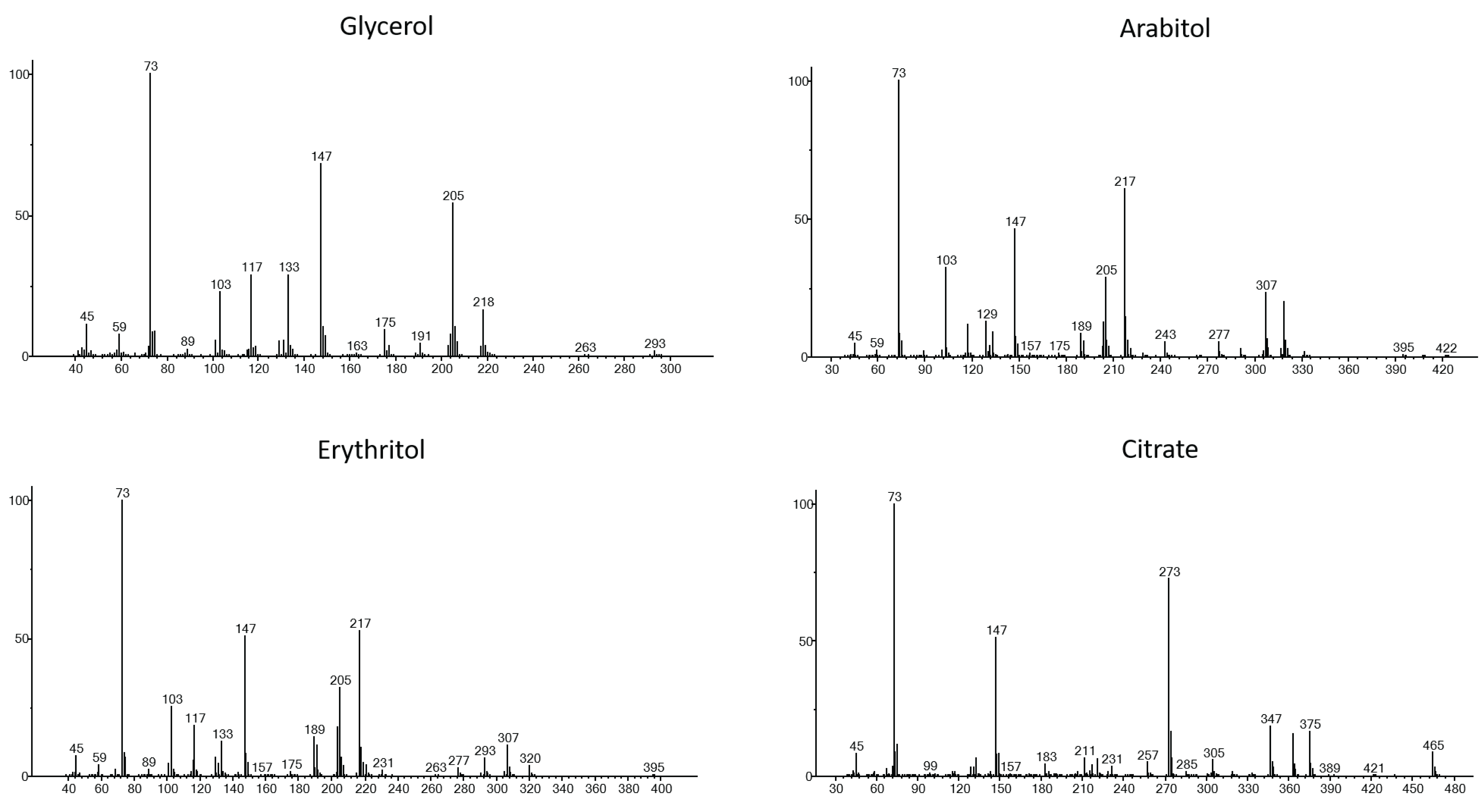

Supplement: Supplementary file 4 — Additional file 4:Figure S4. Gas chromatography-mass spectrometry analysis of sample peaks. Extracted mass spectra for glycerol, arabitol, erythritol, and citric acid. [file 13068_2021_2039_MOESM4_ESM.tif]

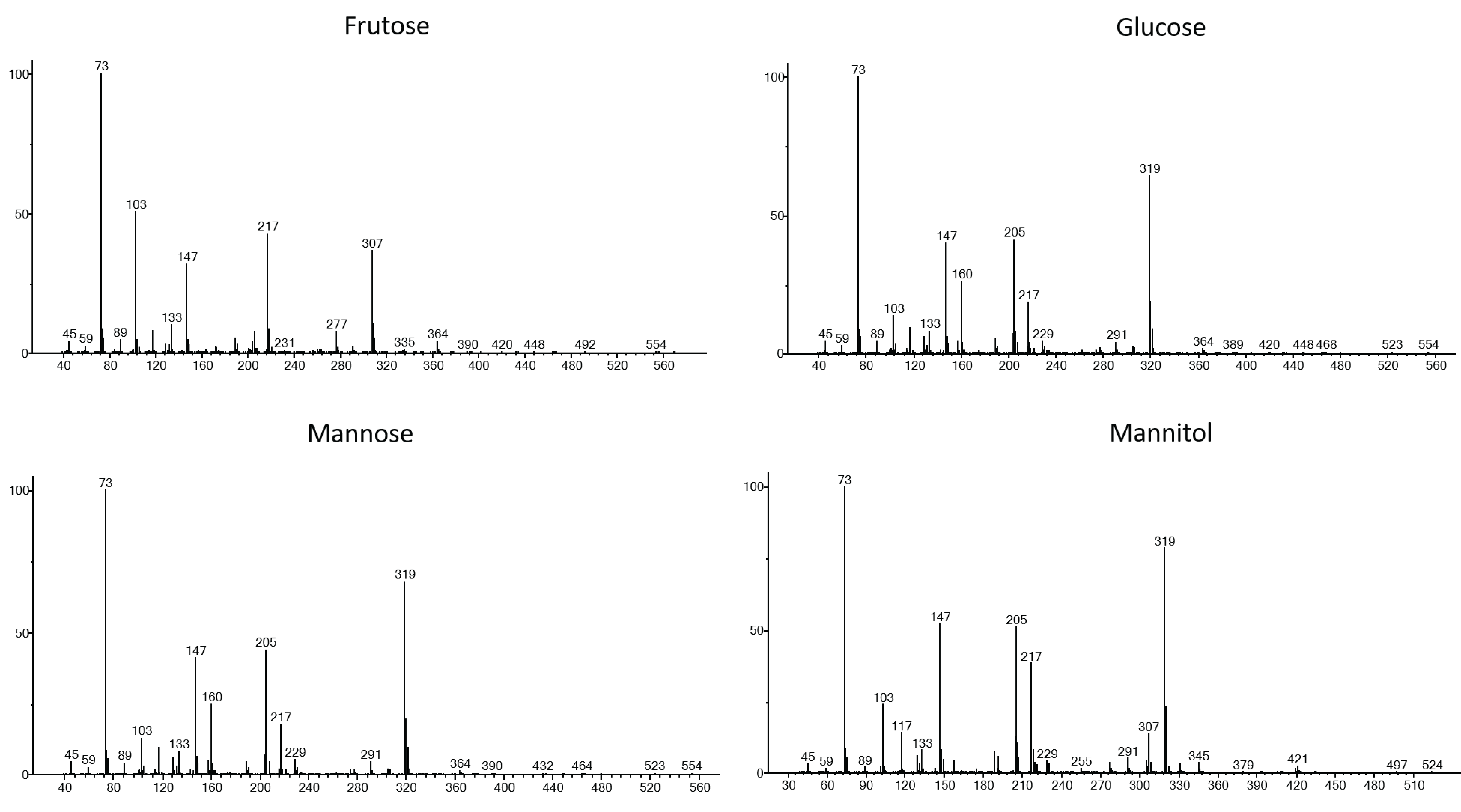

Supplement: Supplementary file 5 — Additional file 5:Figure S5. Gas chromatography-mass spectrometry analysis of sample peaks. Extracted mass spectra for fructose, glucose, mannose, and mannitol. [file 13068_2021_2039_MOESM5_ESM.tif]

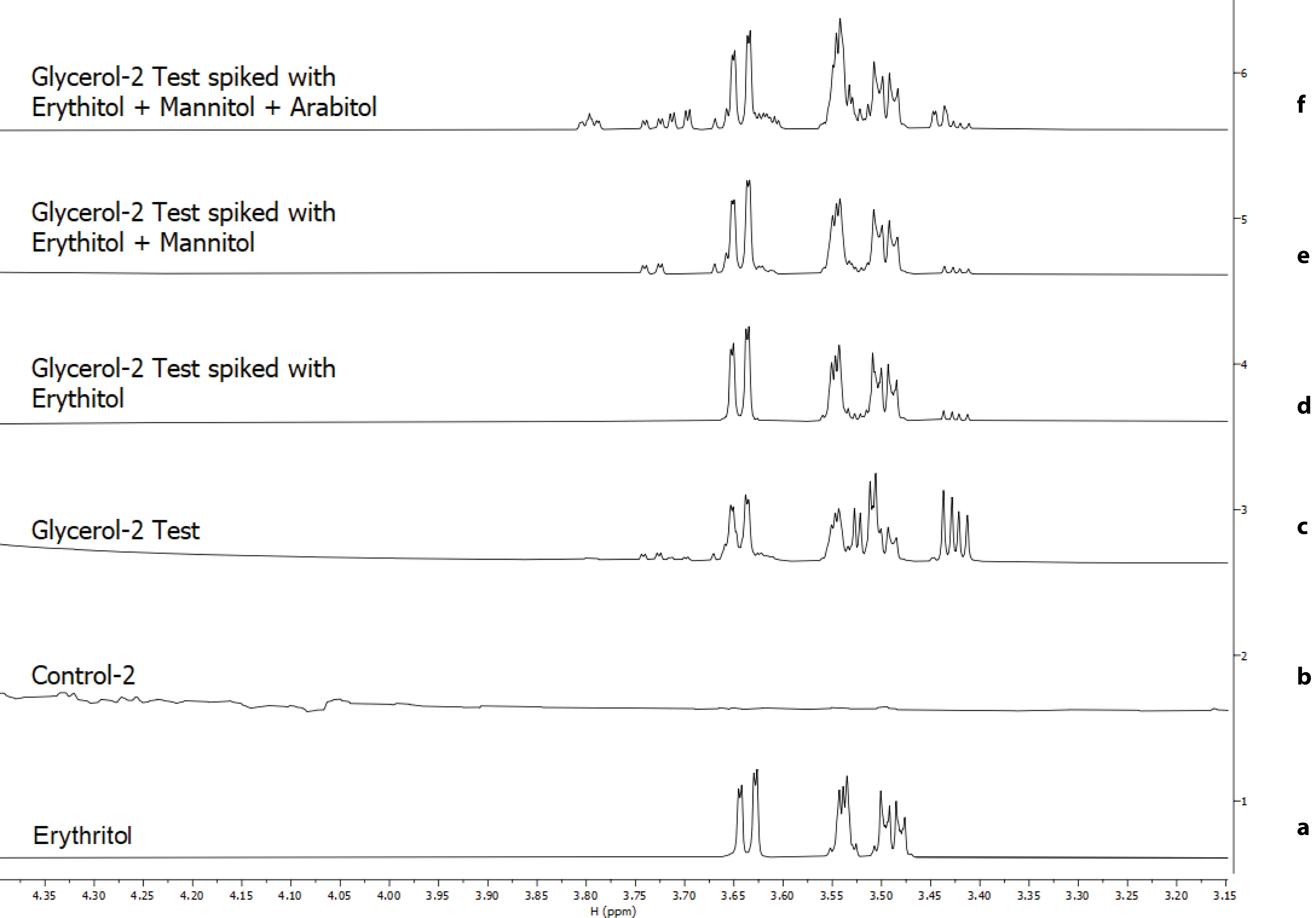

Supplement: Supplementary file 6 — Additional file 6:Figure S6. Proton nuclear magnetic resonance (1H-NMR) spectroscopy analysis of sample peaks. A total of 6 spectra are shown in Figure (all samples were dissolved in 90% H2O and 10% D2O). Panel (a) contains the 1H spectrum of the substrate glycerol. Panel (b) shows the 1H spectrum of the culture media only. No erythritol and additional metabolite signals were detected. Panel (c) contains the 1H spectrum of the products from glycerol. The signals from erythritol show up clearly in this spectrum. Panel (d) is the spectrum collected after a few mg of erythritol powder was added directly to NMR tube. The signals from erythritol increased significantly, again indicating that the peaks in (d) are from erythritol. Panel (e) is the spectrum collected after a few mg of mannitol powder was added directly to NMR tube. Panel (f) is the spectrum collected after a few mg of arabitol powder were added directly to NMR tube. [file 13068_2021_2039_MOESM6_ESM.tif]

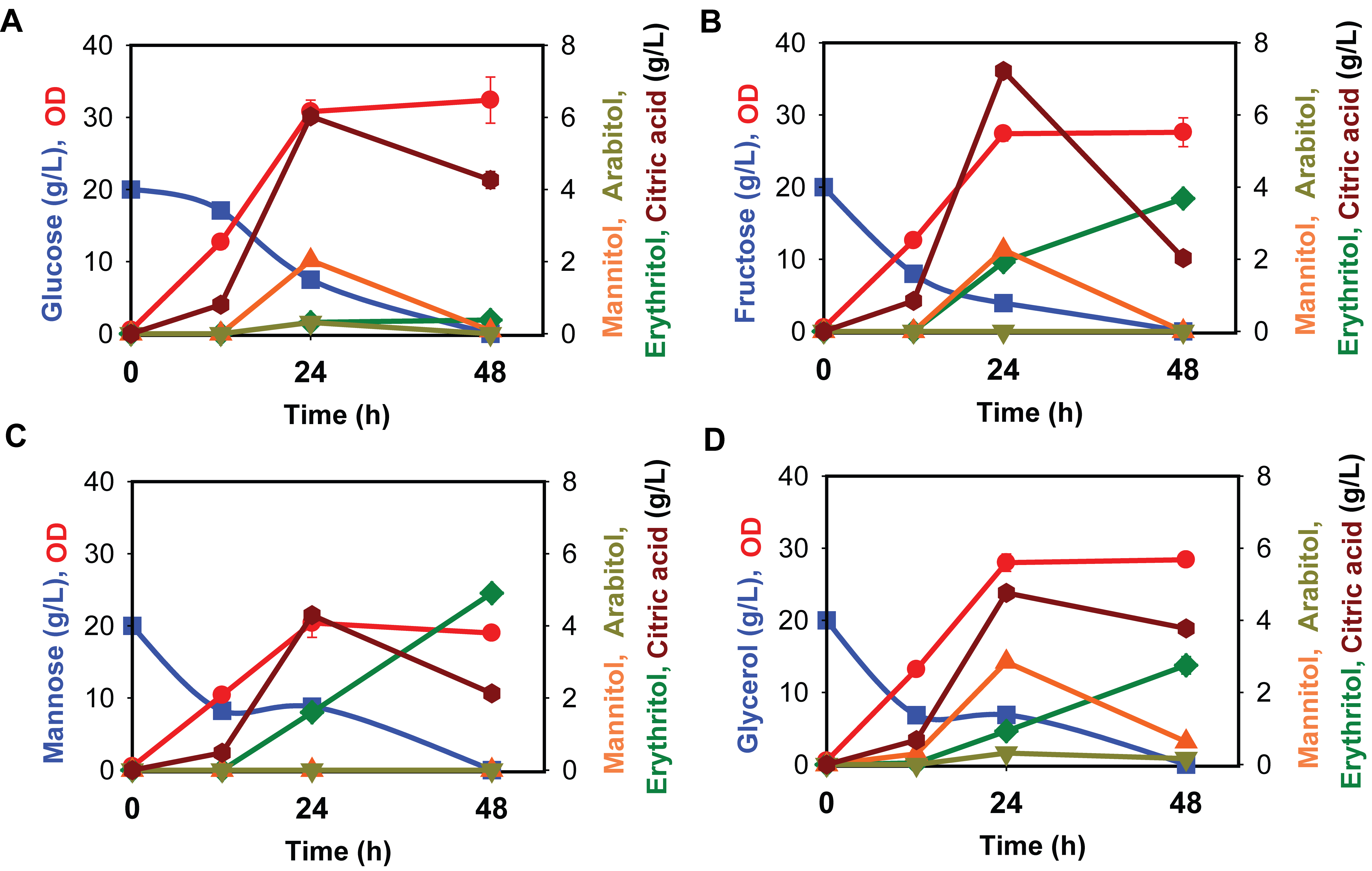

Supplement: Supplementary file 7 — Additional file 7:Figure S7. Growth of Y. lipolytica PO1f on different sugars at 20 g/L in YP medium: (A) glucose, (B) fructose, (C) mannose, and (D) glycerol. [file 13068_2021_2039_MOESM7_ESM.tif]

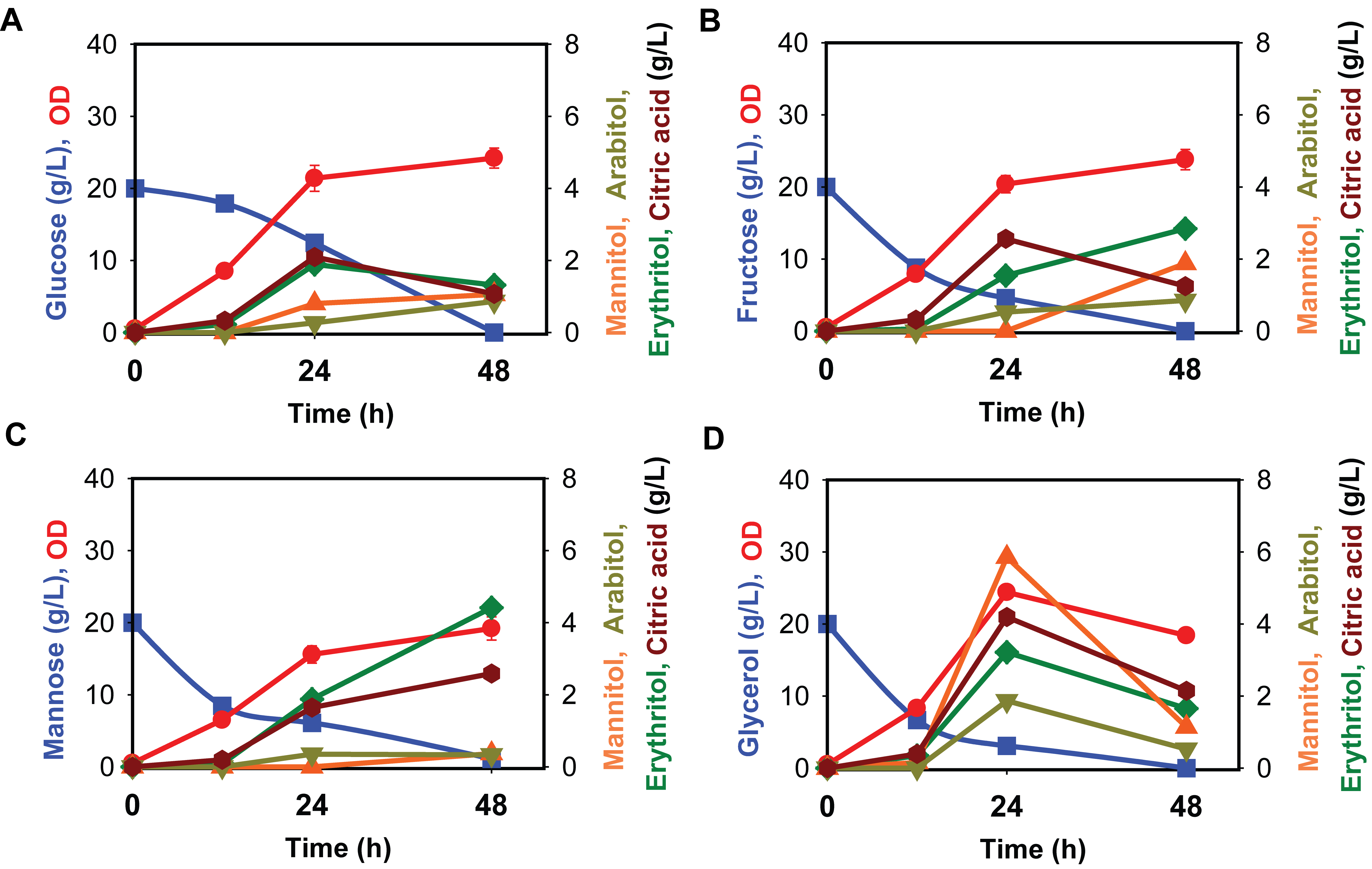

Supplement: Supplementary file 8 — Additional file 8:Figure S8. The effect of osmotic stress (NaCl) on sugar alcohol production in Y. lipolytica PO1f during growth on different sugars in YP medium: (A) glucose, (B) fructose, (C) mannose, and (D) glycerol. Osmotic stress was introduced with the addition of 25 g/L NaCl. [file 13068_2021_2039_MOESM8_ESM.tif]

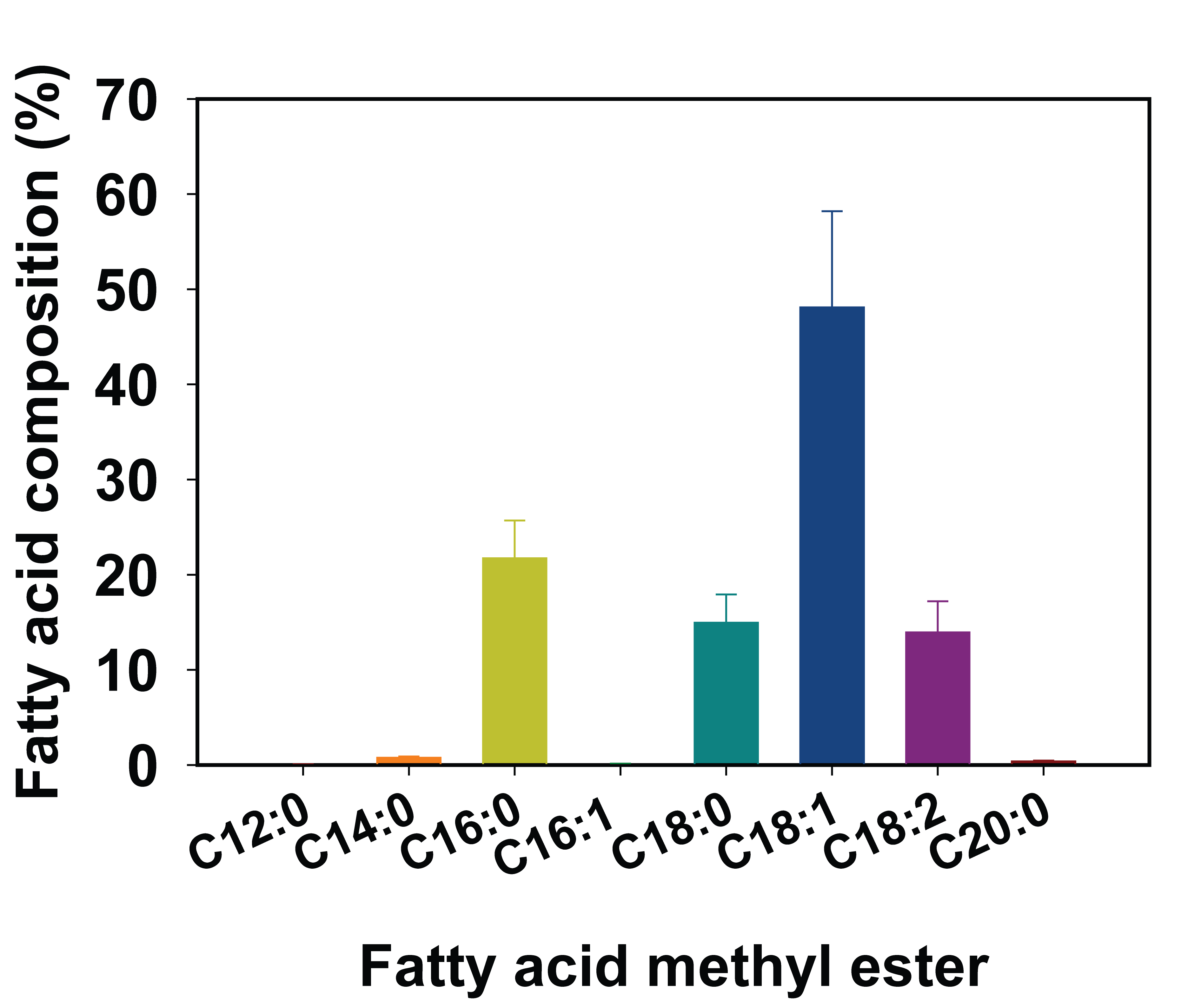

Supplement: Supplementary file 9 — Additional file 9:Figure S9. Fatty acid composition as determined by GC–MS. Data show the mean and standard deviation resulting from two biological and two technical replicates. [file 13068_2021_2039_MOESM9_ESM.tif]

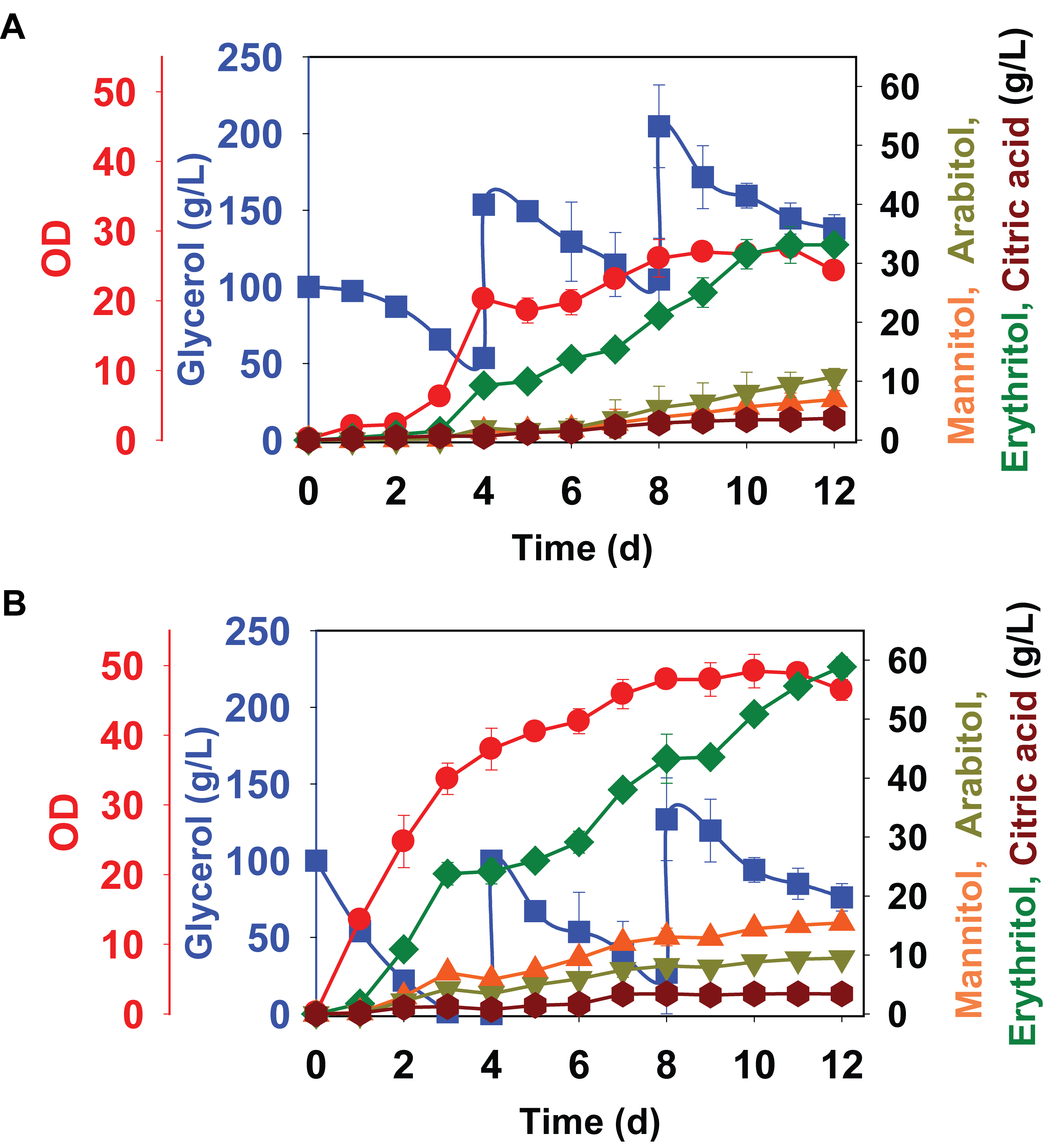

Supplement: Supplementary file 14 — Additional file 14:Figure S10. The intracellular accumulation of erythritol in Yarrowia lipolytica PO1f and Yarrowia lipolytica PO1f-PYP-GK-TKL on glycerol (100 g/L) in PSM medium. [file 13068_2021_2039_MOESM14_ESM.tif]
