# Supplementary material for: Metabolic engineering of the oleaginous yeast Yarrowia lipolytica PO1f for production of erythritol from glycerol
Source: Biotechnol Biofuels. 2021 Sep 25;14:188. doi: 10.1186/s13068-021-02039-0 (PMC8466642; doi:10.1186/s13068-021-02039-0)
Supplement: Supplementary file 10 — Additional file 10:Tables S1. List of genes selected for quantitative PCR. [file 13068_2021_2039_MOESM10_ESM.pdf]

Additional File 10: Tables S1. List of genes selected for quantitative PCR.

	**Protein Name**	**ID**	Gene	**Pathway**
1	Glycerol Kinase	YALI1_F00654p	*GK*	G3P pathway
2	G-3-P dehydrogenase (FAD+)	YALI1_B18499p	*GUT2*	
3	G-3-P dehydrogenase (NAD+)	YALI1_B04433p	*GPD1/GPD2*	
4	Dihydroxyacetone kinase	YALI1_F12917p	*DAK1 / DAK2*	DHA pathway
5	Dihydroxyacetone kinase	YALI1_E24532p	*DAK1 / DAK2*	
6	Glycerol Dehydrogenase (NADP+)	YALI1_B09211p	*GCY1/YPR1*	
7	Glycerol Dehydrogenase (NADP+)	YALI1_B28394p	*GCY1/YPR1*	
8	Arabitol dehydrogenase	YALI1_E15452p	*ADH*	
9	Aldose reductase (NAD(P)+)	YALI1_D09870p	*GRE3*	
10	Aquaglyceroporin	YALI1_F00616p	*FPS1*	Glycerol uptake
11	Aquaglyceroporin	YALI1_E06664p	*FPS1*	
12	Transketolase	YALI1_E07744p	*TKL1*	Pentose phosphate pathway
13	Transaldolase	YALI1_F20914p	*TAL1*	
14	Glucose-6-phosphate dehydrogenase	YALI1_E26811p	*ZWF1*	
15	6-phosphogluconate dehydrogenase	YALI1_B20462p	*GND1*	
16	Actin	Actin 142575p	*ACT*	Internal control
