# Supplementary material for: Metabolic engineering of the oleaginous yeast Yarrowia lipolytica PO1f for production of erythritol from glycerol
Source: Biotechnol Biofuels. 2021 Sep 25;14:188. doi: 10.1186/s13068-021-02039-0 (PMC8466642; doi:10.1186/s13068-021-02039-0)
Supplement: Supplementary file 11 — Additional file 11:Table S2. Oligonucleotides used in this study for qPCR. [file 13068_2021_2039_MOESM11_ESM.pdf]

Additional File 11: Table S2. Oligonucleotides used in this study for qPCR.

Sr. no.	Primer	Sequence
1	jgi|YarliW29_1|6368 FWD Set 1	GAG GCT ACT TGT TTC CAG AC
	jgi|YarliW29_1|6368 REV Set 1	TCC GAG TTG GTC ATT CCT
2	jgi|YarliW29_1|1601 FWD Set 1	AGA ACC TCG AGT CCT CTT AC
	jgi|YarliW29_1|1601 REV Set 1	TCT GGG AGC CAT CAT AGT AG
3	jgi|YarliW29_1|1074 FWD Set 1	GAA GGT CAA CGG CAA GAA
	jgi|YarliW29_1|1074 REV Set 1	CAG GTT GTG AGG AAG TTT GA
4	jgi|YarliW29_1|6827 FWD Set 1	GGA CGC TAT CAA GAA CAA GAA
	jgi|YarliW29_1|6827 REV Set 1	CGG AGA AGA AGA TGG AGT AGA
5	jgi|YarliW29_1|5643 FWD Set 1	GAT TCC CTT CTG TGA AGT CTA C
	jgi|YarliW29_1|5643 REV Set 1	CAT AAC TGG CTC GTC CAT AC
6	jgi|YarliW29_1|1270 FWD Set 1	TGG CTC TCG AGA CAT TGA
	jgi|YarliW29_1|1270 REV Set 1	AAG TTG GAG ACA CCG ATA GA
7	jgi|YarliW29_1|1973 FWD Set 1	GTG GCA TTC TCA CCT CTT AC
	jgi|YarliW29_1|1973 REV Set 1	AGG GTG ACA GCA GAC TTA
8	jgi|YarliW29_1|5256 FWD Set 1	CTC GGA TGT CCA CTA CTA TCT
	jgi|YarliW29_1|5256 REV Set 1	TGA CTT CAG GAC CAA CCT
9	jgi|YarliW29_1|3668 FWD Set 1	GTC ACT GCC TAC TCT TCT TTC
	jgi|YarliW29_1|3668 REV Set 1	TGG TGA TGG TCT CGT TCT
10	YALI1_F00616p FWD Set 1	GAC TCT CTG CTC CTG TAT CT
	YALI1_F00616p REV Set 1	CAG ACC GGT GAT TTC CAT AG
11	YALI1_E06664p FWD Set 1	CTG CCA TCA TCT ACG GAA AC
	YALI1_E06664p REV Set 1	GAG CAG GGT AGG TAC AGA ATA
12	YALI1_E07744p FWD Set 1	GAT ACT CTC ACC AGC AGT TTG
	YALI1_E07744p REV Set 1	CTG GCC CTT GTA GTA GTT AAT G

13	YALI1_F20914p FWD Set 1	AGC ACG GTA TCC ACT GTA
	YALI1_F20914p REV Set 1	CCA ACA AAG GGA GAG ATG AG
14	YALI1_E26811p FWD Set 1	GGA ATC ATC CGA GAC GTT ATT
	YALI1_E26811p REV Set 1	GCA CCT TGA CCT TCT CAT C
15	YALI1_B20462p FWD Set 1	CAT CAT TGA CGG TGG TAA CT
	YALI1_B20462p REV Set 1	CAC CGG AAC CAA CAA AGA
16	Actin 142575p FWD Set 1	AAG TCC AAC CGA GAG AAG A
	Actin 142575p REV Set 1	CAC CAG AGT CAA GAA CGA TAC
