# Supplementary material for: Metabolic engineering of the oleaginous yeast Yarrowia lipolytica PO1f for production of erythritol from glycerol
Source: Biotechnol Biofuels. 2021 Sep 25;14:188. doi: 10.1186/s13068-021-02039-0 (PMC8466642; doi:10.1186/s13068-021-02039-0)
Supplement: Supplementary file 12 — Additional file 12:Table S3. A total of 68 intracellular metabolites such as amino acids, sugars, phosphates, fatty acids, organic acids, nucleosides and others, identified under glycerol without NaCl and glycerol with NaCl conditions by GC–MS analysis in Y. lipolytica Po1f. [file 13068_2021_2039_MOESM12_ESM.pdf]

Additional File 12: Table S3. A total of 68 intracellular metabolites such as amino acids, sugars, phosphates, fatty acids, organic acids, nucleosides and others, identified under glycerol without NaCl and glycerol with NaCl conditions by GC-MS analysis in *Y. lipolytica* Po1f.

Sr. no.	Metabolite	relative concentration			
		Without NaCl		With NaCl	
		Average	STDE	Average	STDE
1	E-15-Heptadecenal	131.17	19.10	73.18	5.07
2	1-Monohexadecanoylglycerol	16.00	0.90	17.02	2.71
3	2-Isopropylmalic acid	212.36	35.24	756.21	115.78
4	GABA	64.04	1.29	46.59	6.61
5	4-Hydroxyproline	0.00	0.00	5.08	0.23
6	5-Methylthioadenosine	0.00	0.00	15.35	1.40
7	C18:2 (9,12)	180.88	0.87	148.37	0.27
8	C16:1 (9)	25.78	6.34	33.79	0.54
9	α-Ketoglutaric acid	34.18	13.16	61.78	4.60
10	Alanine	414.39	144.79	260.89	33.55
11	Aminomalonic acid	19.97	8.11	0.00	0.00
12	Arabitol	99.13	14.66	1063.13	207.15
13	Arginine	0.00	0.00	2.17	0.60
14	Aspartic acid	20.73	3.89	62.60	16.37
15	Benzoic acid	1043	154.74	564.35	42.70
16	C18:1 (11)	21.86	5.87	10.36	0.87
17	C18:1 (9)	174.45	64.13	75.33	27.73

18	Cadaverine	0.00	0.00	6.74	1.72
19	Citric acid	0.00	0.00	4.72	0.07
20	Diethyleneglycol	9.58	2.45	6.79	1.18
21	Diglycerol	0.00	0.00	4.81	1.86
22	C22:0	52.50	9.61	29.95	3.16
23	Docosanol	26.01	4.01	14.41	1.03
24	C20:0	20.96	2.72	10.89	0.99
25	Ergosterol	51.78	10.69	36.67	8.28
26	Erythritol	881.17	12.81	9629.94	2171.22
27	Ethanolamine	74.72	21.83	102.71	18.35
28	Ethanolamine glycerophosphate	9.78	3.39	47.85	5.53
29	Fructose	49.03	6.67	77.34	9.15
30	Fumaric acid	5.00	0.75	7.57	1.37
31	Glucose	27.06	1.00	32.85	4.59
32	Glutamic acid	4.67	1.00	114.22	0.26
33	Glutamine	125.00	35.81	337.47	39.92
34	Glycerol	32286.63	4532.00	35414.65	7532.83
35	Glycerol-3-p	25.19	0.08	19.17	0.05
36	Glycine	55.85	4.80	109.14	18.31
37	Glycolic acid	22.07	5.07	14.65	3.07
38	Glyoxylic acid	32.11	2.56	15.45	0.75
39	Inositol, myo	20.58	3.36	66.34	6.79
40	Isoleucine	0.00	0.00	27.77	2.00
41	lactic acid	86.70	18.96	145.20	78.34
42	Leucine	1.28	0.00	2.79	0.39
43	Lysine	0.00	0.00	34.16	6.04
44	Malic acid	0.00	0.00	3.10	0.34

45	Mannitol	1031.25	11.94	2683.60	352.35
46	N-α-Acetyl-L-Lysine	0.00	0.00	0.00	0.00
47	N-Acetylglutamic acid	44.11	11.11	43.21	3.36
48	Octadecane	38.51	8.61	20.31	4.98
49	Octadecanol	35.02	7.30	17.86	2.26
50	Palmitic acid	2463.83	583.81	1302.93	203.08
51	Phenylalanine	3.35	0.29	10.72	3.18
52	Proline	6.93	1.99	36.97	3.52
53	Putrescine	19.94	13.21	51.76	15.85
54	pyruvic acid	122.07	13.65	166.47	41.68
55	Ribitol	6.38	0.39	49.13	10.46
56	Ribose	5.81	3.06	22.12	3.52
57	Serine	19.79	3.69	42.15	0.67
58	sorbitol	19.19	1.02	20.53	1.99
59	Spermidine	14.05	2.93	576.57	171.77
60	Stearic acid	2629.80	558.44	1600.21	210.43
61	Succinic acid	37.96	6.63	45.19	7.02
62	Tetradecanoic acid	47.02	8.49	27.86	2.83
63	Threitol	0.00	0.00	4.35	1.48
64	Threonine	53.66	11.07	85.25	2.80
65	Trehalose	39.83	1.97	11.30	1.07
66	Tryptophan	0.00	0.00	0.52	0.11
67	Tyrosine	0.00	0.00	9.68	0.84
68	Valine	20.07	4.62	26.15	0.69
