# Supplementary material for: Metabolic engineering of the oleaginous yeast Yarrowia lipolytica PO1f for production of erythritol from glycerol
Source: Biotechnol Biofuels. 2021 Sep 25;14:188. doi: 10.1186/s13068-021-02039-0 (PMC8466642; doi:10.1186/s13068-021-02039-0)
Supplement: Supplementary file 13 — Additional file 13:Table S4. Nucleotide sequence of codon optimized sugar alcohol phosphatase. [file 13068_2021_2039_MOESM13_ESM.pdf]

**Additional File 13: Table S4**. Nucleotide sequence of codon optimized sugar alcohol phosphatase.

| Name | Sequence (5’-3’) |
|---|---|
| *PYP* | gaatcattcaaaggcgcgccATGGTCAAGGCTGTGATCTTCACGGATTTCGATG<br>GCACTGTCACTCTGGAGGACAGCAACGATTACCTTACAGATACACTTG<br>GTTTTGGAAAAGAGAAACGGCTCAAGGTTTTCGAAGGCGTTCTGGATG<br>ACACCAAATCGTTCCGTCAAGGTTTCATGGAAATGCTTGAGTCGATTC<br>ATACACCTTTCCCTGAATGCATTAAAATTCTGGAGAAAAAGATTAGACT<br>TGATCCCGGCTTCAAAGATACATTTGAGTGGGCACAGGAAAATGATGT<br>CCCTGTTATTGTCGTTAGCTCTGGAATGAAACCCATTATCAAGGTCCTT<br>CTGACCCGACTTGTCGGTCAGGAATCCATCCATAAAATCGACATTGTT<br>TCGAACGAAGTGGAGATCGATGCACACGACCAGTGGAAGATCATTTAC<br>AAAGACGAAAGCCCTTTTGGACACGATAAAAGCCGATCCATTGATGCT<br>TACAAAAAAAAGTTCGAATCGACGCTGAAGGCAGGCGAGCAGAGACC<br>TGTTTATTTTTACTGTGGAGATGGAGTCTCCGATCTCTCTGCAGCAAAA<br>GAATGTGATCTCCTGTTCGCAAAGCGGGGCAAAGATCTGGTTACCTAT<br>TGCAAGAAGCAGAATGTCCCCTTCCATGAGTTTGACACGTTCAAGGAC<br>ATCCTGGCCTCGATGAAACAAGTGCTGGCTGGAGAAAAAACCGTTGCT<br>GAGCTTATGGAGAACTAGgctagcctcatgtaattag |
