# Supplementary material for: Metabolic engineering of the oleaginous yeast Yarrowia lipolytica PO1f for production of erythritol from glycerol
Source: Biotechnol Biofuels. 2021 Sep 25;14:188. doi: 10.1186/s13068-021-02039-0 (PMC8466642; doi:10.1186/s13068-021-02039-0)
Supplement: Supplementary file 15 — Additional file 15:Table S5. Oligonucleotides used in this study. [file 13068_2021_2039_MOESM15_ESM.pdf]

Additional File 15: Table S5. Oligonucleotides used in this study.

Primer	Sequence	Characteristic
Sjpyp co yl FP	CAT TCA AAG GCG CGC CAT GGT CAA GGC TGT G	PYP PCR
Sjpyp co yl RP	CAT GAG GCT AGC CTA GTT CTC CAT AAG C	PYP PCR
SjTKL yl FP	gaatcattcaaaggcgcgccATG GCT CCC CAA TTT TCA AAG ACT GAC G	TKL PCR
SjTKL yl RP	CTA ATT ACA TGA GGC TAG CTT AGA CAC CGT GGC CGG GTC	TKL PCR
SjGK yl FP	gaatcattcaaaggcgcgccATG TCT TCC TAC GTA GGA GCT CTC GAC	GK PCR
SjGK yl RP	CTA ATT ACA TGA GGC TAG CCTG GTC GAG AGC TCC TAC GTA GGA AG	GK PCR
SJ Pyp1 YL M FP	CTG GAG GAC AGC AAC GAT TAC CTT ACA GAT AC	PYP middle PCR
SJ Pyp1 YL M RP	CAG GAT GTC CTT GAA CGT GTC AAA CTC ATG	PYP middle PCR
SJ GK YL FP	gaatcattcaaa AAGCTTATGTCTTCCTACGTAGGAGCTCTCGA	GK middle PCR
SJ GK YL RP	CTA ATT ACA TGA GGG CGC GCC TTA CTC AAG CCA GCC AAC AGC TC	GK middle PCR
pCHPH FP	GCC ACG GTG TCT AAC TCG AGT CAT GTA ATT AGT TAT GTC ACG	pCHPH Gibson PCR
pCHPH RP	GGA GCC ATG CTG CGG TTA GTA CTG C	pCHPH Gibson PCR
TKL FP	gcagtactaaccgcagcATGGCTCCCCAATTTTCAAAGACTGAC	TKL Gibson PCR
TKL RP	ATG ACT CGA GTT AGA CAC CGT GGC CGG GTC	TKL Gibson PCR
TKL check FP	ATGGCTCCCCAATTTTCAAAGACTGAC	TKL check PCR
TKL middle check RP	TCG TCG GAT GAT CTC AGA GTG CTC	TKL check PCR
TKL check RP	TT AGA CAC CGT GGC CGG GTC	TKL check PCR
GK YL check FP	ATGTCTTCCTACGTAGGAGCTCTCGA	GK check PCR
GK YL middle check RP	GCTC TCC AAT CTG CTC GGA AGA CTC	GK check PCR
GK YL check RP	GAGCTGTTGGCTGGCTTGAGTAA	GK check PCR
USB16 middle FP	CTT CTC TTT GTG TGT AGT GTA CGT ACA TTA TC	PYP check PCR

GK middle RP	GAG GGT CTC AAT GTT CAT GAA CAT G	GK check PCR
TEFintr middle FP	GAA TCT ACG CTT GTT CAG ACT TTG TAC TAG	TKL check PCR
TKL middle RP	TCA ATA GTC TTG GCG TAG AGA TCA G	TKL check PCR
SJ GK YL FP M	gaatcatAAGCTT ATGTCTTCCTACGTAGGAGCTCTCGA	GK check PCR
SJ GK YL RP M	T ACA TGA GGG CGC GCC TTA CTC AAG CCA GCC AAC AGC TC	GK check PCR
